# Dynamic associations among exercise self-efficacy, extracurricular exercise involvement, and perceived physical fitness in Chinese undergraduates: a three-wave RI-CLPM study

**DOI:** 10.3389/fpsyg.2026.1909123

**Published:** 2026-09-29

**Authors:** Yihe Gai, Shengli Duan, Meng Dai

**Affiliations:** 1 Hongyi College, Chengdu Technological University, Chengdu, China; 2 School of Physical Education and Greater Health, Sichuan Technology and Business University, Chengdu, Sichuan, China; 3 Faculty of Education, Shinawatra University, Pathum Thani, Thailand

**Keywords:** exercise self-efficacy, extracurricular exercise involvement, longitudinal study, perceived physical fitness, physical activity, random-intercept cross-lagged panel model, university students

## Abstract

**Inroduction:**

University students often experience difficulties maintaining regular extracurricular exercise, yet less is known about how exercise-related confidence, involvement, and perceived fitness unfold within individuals over time. This three-wave longitudinal study examined the dynamic associations among Exercise Self-Efficacy (ESE), Extracurricular Exercise Involvement (EEI), and Perceived Physical Fitness (PPF) in Chinese undergraduates using a random-intercept cross-lagged panel model (RI-CLPM).

**Methods:**

Undergraduate students aged 18 years or older from eight universities in Sichuan Province, China, participated in offline surveys at three waves with approximately eight-week intervals. The baseline (T1) analytic sample included 1,673 students; 1,543 provided valid follow-up data at T2, and 1,455 provided valid follow-up data at T3. Manifest mean scores of ESE, EEI, and PPF were analyzed in Mplus 8.3 using robust maximum likelihood and full-information maximum likelihood for missing data.

**Results:**

The free-lag RI-CLPM showed excellent fit and was retained as the final model. At the within-person level, higher-than-usual ESE predicted higher-than-usual EEI at the subsequent wave across both intervals. Higher-than-usual EEI also predicted higher-than-usual PPF across both intervals. In addition, EEI at T2 positively predicted ESE at T3; individual between-interval path differences did not survive multiplicity adjustment. In contrast, PPF did not significantly predict later ESE or EEI. The specific indirect association from ESE_T1 to PPF_T3 through EEI_T2 was small (b = 0.02250; standardized estimate = 0.02038) and received tentative support from the 95% bias-corrected bootstrap interval [0.004, 0.051], while the Wald test was marginal (*p* = 0.055).

**Discussion:**

These findings identify exercise self-efficacy and the quality of extracurricular exercise involvement as candidate targets for experimental research on university students’ perceived fitness.

## 1 Introduction

Physical activity and exercise are widely recognized as essential foundations of health across the life span. The World Health Organization recommends that adults accumulate 150–300 min of moderate-intensity aerobic physical activity, or 75–150 min of vigorous-intensity aerobic physical activity, per week for substantial health benefits (Bull et al., 2020). For university students, however, maintaining regular exercise is often challenging. The transition into university is accompanied by greater autonomy, academic pressure, irregular schedules, and changes in social routines, all of which may weaken students’ capacity to maintain an active lifestyle. Earlier and recent reviews have consistently shown that many university students do not engage in sufficient physical activity to obtain health benefits (Brown et al., 2024; Irwin, 2004). In China, a systematic review similarly indicated that college students’ exercise behavior is shaped by multiple constraints, including academic pressure, lack of time, limited facilities, and insufficient professional guidance (Pan et al., 2022). These findings suggest that promoting exercise among undergraduates requires more than increasing opportunities for participation; it also requires understanding the psychological and behavioral processes through which students become confident, involved, and physically self-aware (Babaeer et al., 2022; Dziewior et al., 2024; Guthold et al., 2018; Guthold et al., 2020; Pengpid et al., 2015; Pirwani and Szabo, 2024; Pirwani and Szabo, 2025; Santos et al., 2023; Strain et al., 2024; Warburton and Bredin, 2017; World Health Organization, 2022; Zhang et al., 2023).

A key conceptual issue in this field is that exercise behavior should not be reduced to frequency, duration, or intensity alone. Although such indicators are important, they may not fully capture the quality of students’ engagement in exercise. Following the classic distinction among physical activity, exercise, and physical fitness, exercise can be understood as planned, structured, and purposeful physical activity aimed at maintaining or improving fitness (Caspersen et al., 1985). In university settings, extracurricular exercise is particularly important because it reflects students’ voluntary participation outside compulsory physical education classes or organized training. However, two students who exercise with similar frequency may differ substantially in their persistence, concentration, perceived value, and autonomy during exercise. Therefore, the present study focuses on Extracurricular Exercise Involvement (EEI), defined as students’ active, persistent, autonomous, and immersive psychological involvement in voluntary exercise outside compulsory physical education or formal sport training. This construct extends beyond behavioral participation and reflects the motivational and experiential quality of exercise. Dong (2017) conceptualized physical exercise involvement among Chinese college students as a positive psychological state characterized by vigor-persistence, absorption and satisfaction, value cognition, and participation autonomy. The measure assesses the motivational and experiential quality of engagement; it does not quantify exercise frequency, duration, intensity, or energy expenditure. Thus, EEI offers complementary information to exercise frequency for understanding why some students maintain exercise and experience positive physical outcomes (Bauman et al., 2012; Newsome et al., 2023; Van Luchene and Delens, 2021).

The first theoretical construct in the present model is Exercise Self-Efficacy (ESE). Based on social cognitive theory, self-efficacy refers to individuals’ beliefs about their capability to organize and execute actions required to attain desired outcomes (Bandura, 1997). In the context of exercise, ESE reflects students’ confidence in their ability to initiate and maintain exercise despite barriers such as fatigue, limited time, negative mood, academic workload, or lack of social support. Social cognitive theory proposes that self-efficacy is a central mechanism through which personal, behavioral, and environmental influences regulate motivation and action (Bandura, 2004). In young adults, prospective evidence has shown that self-efficacy is a key social-cognitive determinant of later physical activity, partly through self-regulatory processes (Rovniak et al., 2002). Theoretically, students with higher ESE should be more likely to transform exercise intentions into actual involvement because they are more confident in overcoming barriers and sustaining effort. At the same time, successful exercise experiences may provide mastery feedback and thereby strengthen subsequent self-efficacy. This possibility highlights a potentially reciprocal process between ESE and EEI rather than a purely one-directional association (Ashford et al., 2010; Bao, 2025; Bateman et al., 2022; Chen and Xiang, 2025; Gao et al., 2025; Sheeran et al., 2016; Williams and French, 2011; Young et al., 2014).

Social cognitive theory and self-determination theory offer complementary accounts of this prospective association. ESE concerns an anticipatory judgment about the ability to meet exercise demands, whereas competence need satisfaction describes the experienced sense of effectiveness and growth during an activity (Bandura, 1997; Deci and Ryan, 2000). Stronger capability beliefs may reduce the perceived costs of fatigue, embarrassment, or unsuccessful performance, leaving more attention available for persistence, absorption, and enjoyment. When activities also provide meaningful choice and manageable challenges, these experiences may support autonomous engagement and personal value (Teixeira et al., 2012). ESE can therefore be considered a resource for the quality of subsequent involvement. Competence satisfaction may help explain this link, although it is conceptually distinct from ESE and was not measured in the present study.

The third focal construct in the present study is Perceived Physical Fitness (PPF). Physical fitness represents the capacity of body systems to support daily activity, exercise, and health-related functioning. It is not merely an athletic attribute but an important health marker, particularly during youth and young adulthood (Ortega et al., 2008). Objective fitness testing is valuable but is often difficult to implement repeatedly in large-scale longitudinal surveys because it requires equipment, trained personnel, standardized testing conditions, and substantial participant time. For this reason, perceived fitness has become a practical and meaningful complementary indicator. The International Fitness Scale was developed to assess self-reported fitness across general fitness, cardiorespiratory fitness, muscular strength, speed/agility, and flexibility, and evidence supports its usefulness for ranking young people according to fitness levels and identifying health-related differences (Ortega et al., 2011). Chinese evidence also suggests that the International Fitness Scale shows moderate reliability among Chinese children and adolescents, while further validation in Chinese subgroups remains desirable (Bao et al., 2022). In the present study, PPF is defined as students’ subjective evaluation of their overall and domain-specific physical fitness. PPF is conceptualized as a self-evaluative health perception that may both reflect previous exercise experiences and influence later exercise confidence and involvement (Ma et al., 2024; Ruiz et al., 2009; Wang, 2019; Yang et al., 2024).

PPF may occupy two positions in this process: a self-evaluative outcome of previous exercise experiences and a potential source of information for subsequent confidence and involvement. The feedback role may depend on how perceived bodily changes are interpreted. Improvements attributed to personal effort or increasing capability could reinforce ESE, whereas comparisons with fitter peers or temporary fluctuations in energy may provide less consistent guidance for future exercise. Accordingly, positive feedback from PPF is theoretically plausible but may vary with the appraisal context and the interval over which it is observed (Bandura, 2004; Ortega et al., 2011).

Integrating these constructs, the present study proposes a dynamic process linking ESE, EEI, and PPF. From a social cognitive perspective, students who feel more capable of exercising are more likely to invest effort, persist in the face of obstacles, and experience greater involvement in extracurricular exercise. In turn, stronger exercise involvement may lead students to perceive improvements in their physical fitness because sustained and meaningful engagement provides repeated bodily feedback, mastery experiences, and opportunities to notice changes in endurance, strength, flexibility, and general physical functioning. Therefore, the central theoretical pathway is that higher ESE is prospectively associated with EEI, which in turn is associated with later PPF. This pathway is theoretically important because it links a capability belief, the quality of exercise engagement, and a later self-evaluative outcome (Huang et al., 2024; Plotnikoff et al., 2015; Rhodes and Kates, 2015; Teixeira et al., 2012; Yuan et al., 2024).

Nevertheless, the association among these constructs is unlikely to be exclusively unidirectional. Social cognitive theory emphasizes reciprocal determinism, meaning that personal beliefs, behavior, and perceived outcomes can mutually influence one another over time (Bandura, 2004). For example, students who become more involved in extracurricular exercise may later develop stronger ESE because repeated exercise experiences create mastery and reduce perceived barriers. Similarly, students who perceive themselves as physically fitter may become more willing to exercise and more confident in their ability to maintain exercise. Thus, the relationship among ESE, EEI, and PPF may represent a positive developmental cycle in which exercise confidence promotes involvement, involvement strengthens perceived fitness, and perceived fitness feeds back into later confidence and involvement. Testing this dynamic cycle is important for moving beyond static explanations of college students’ exercise behavior (Deng et al., 2023; Hu and Liu, 2025; Sheng et al., 2025; Taylor et al., 2025; Zhou et al., 2025).

A major limitation of previous research is that much of the evidence linking exercise-related self-efficacy, exercise behavior, and perceived fitness has been cross-sectional. Cross-sectional mediation models can estimate whether variables are statistically associated, but they cannot determine whether changes in one construct precede changes in another. Even traditional cross-lagged panel models may be insufficient when stable individual differences are present. Hamaker et al. (2015) argued that conventional cross-lagged panel models may confound stable between-person differences with within-person processes, leading to misleading interpretations of longitudinal associations. This distinction is crucial in the present context. Students who are generally more confident may also be generally more involved and perceive themselves as fitter, but this does not necessarily mean that a temporary increase in confidence within the same student will lead to later increases in involvement or perceived fitness. The present study uses a random-intercept cross-lagged panel model (RI-CLPM). The RI-CLPM separates stable between-person differences from within-person fluctuations, allowing cross-lagged paths to be interpreted as whether deviations from a student’s own usual level predict later deviations in another construct (Hamaker et al., 2015; Mulder and Hamaker, 2021; Little, 2013; Orth et al., 2021; Usami, 2021; Zheng and Valente, 2023).

Using a three-wave longitudinal design, the present study examines the dynamic associations among ESE, EEI, and PPF in Chinese undergraduates. Specifically, the study aims to determine whether students who report higher-than-usual ESE at one wave subsequently report higher-than-usual EEI, and whether higher-than-usual EEI subsequently predicts higher-than-usual PPF. The study also examines competing and feedback pathways, including whether EEI predicts later ESE and whether PPF predicts later ESE or EEI. By separating between-person differences from within-person change, this study seeks to clarify whether the proposed associations occur within individuals after accounting for stable differences between students.

Taken together, the present study used a three-wave longitudinal design and a random-intercept cross-lagged panel model to examine the dynamic associations among Exercise Self-Efficacy (ESE), Extracurricular Exercise Involvement (EEI), and Perceived Physical Fitness (PPF) in Chinese undergraduates. At the between-person level, higher average levels of one construct were expected to be associated with higher average levels of the other constructs across the study period (H1). At the within-person level, it was hypothesized that occasions on which students reported higher-than-usual ESE would be followed by higher-than-usual EEI at the subsequent wave (H2), and that higher-than-usual EEI would be followed by higher-than-usual PPF at the subsequent wave (H3). A positive three-wave longitudinal indirect pathway from ESE at Time 1 to PPF at Time 3 through EEI at Time 2 was also expected (H4). Consistent with the reciprocal assumptions of social cognitive theory, reverse and feedback paths among ESE, EEI, and PPF were estimated as theoretically meaningful competing pathways within the full dynamic model (H5). The proposed theoretical model, including the primary hypothesized pathway and the additional autoregressive and cross-lagged paths estimated in the full RI-CLPM, is presented in Figure 1.

**Figure 1:**
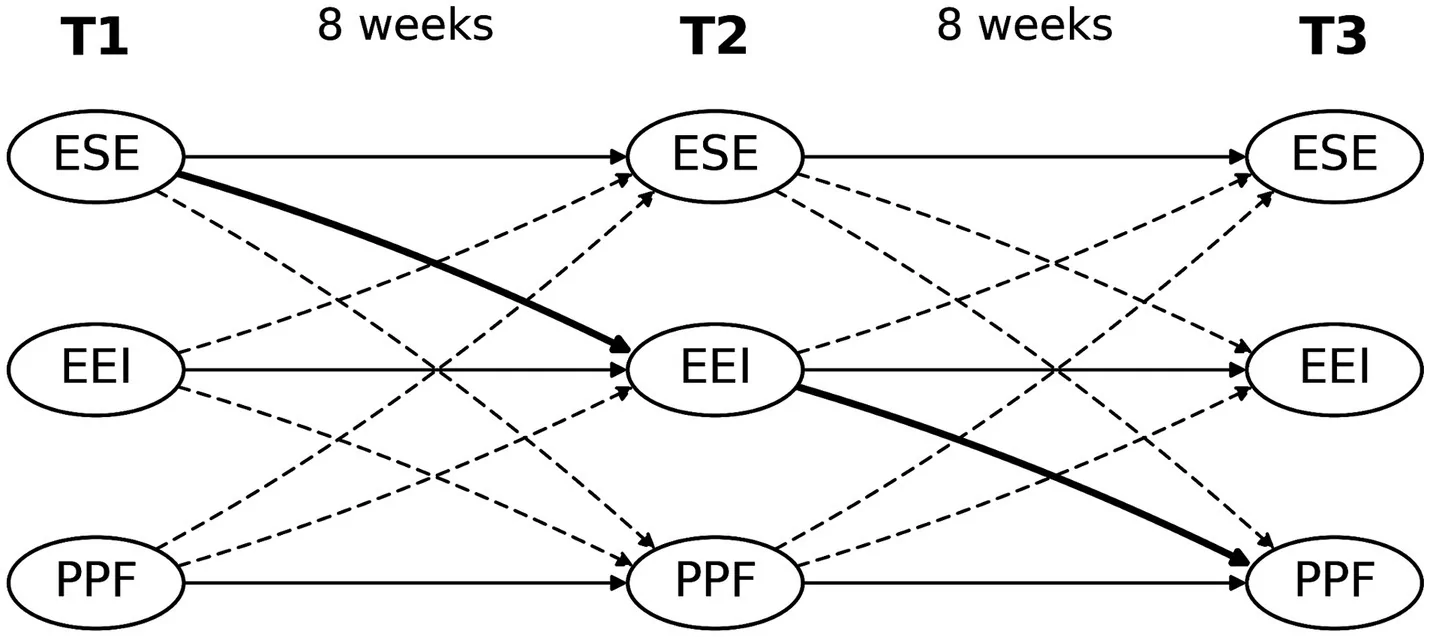
Proposed three-wave RI-CLPM of exercise self-efficacy, extracurricular exercise involvement, and perceived physical fitness. ESE, exercise self-efficacy; EEI, extracurricular exercise involvement; PPF, perceived physical fitness; RI-CLPM, random-intercept cross-lagged panel model. T1, T2, and T3 denote the three measurement occasions. Thick solid arrows identify ESE_T1 → EEI_T2 → PPF_T3, the focal hypothesized sequence. Thin solid arrows indicate autoregressive paths, and dashed arrows indicate the other cross-lagged paths estimated in the full model. Line types identify theoretical path roles, not statistical significance. Nodes represent the within-person components conceptually; random intercepts, same-wave covariances, and innovation covariances are omitted for clarity.

## 2 Materials and methods

### 2.1 Participants and procedure

This study used a three-wave longitudinal survey design to examine the dynamic associations among exercise self-efficacy, extracurricular exercise involvement, and perceived physical fitness in Chinese undergraduates. Participants were recruited from eight universities in Sichuan Province, China: Chengdu Technological University, Southwest Petroleum University, Sichuan University, University of Electronic Science and Technology of China, Southwestern University of Finance and Economics, Southwest Jiaotong University, Civil Aviation Flight University of China, and Xihua University. A multisite convenience sampling strategy was used. Eligible participants were full-time undergraduate students who were at least 18 years old, were able to complete a Chinese-language questionnaire independently, and provided written informed consent before participation. Students younger than 18 years, students who declined informed consent, and questionnaires that failed the data-quality screening criteria were excluded.

The study protocol was reviewed and approved by the Ethics Committee of Hongyi College, Chengdu Technological University, China (approval number: CDTU2025TY014). All procedures were conducted in accordance with the ethical principles of the Declaration of Helsinki for research involving human participants (World Medical Association, 2024). Before completing the questionnaire, participants received a written information sheet describing the purpose of the study, the voluntary nature of participation, confidentiality protections, potential risks and benefits, and their right to withdraw at any time without penalty. The first page of the questionnaire explicitly stated that only adults aged 18 years or older were eligible to participate. Written informed consent was obtained from all participants before data collection.

Data were collected at three waves with approximately eight-week intervals. The first wave was conducted from March 3 to March 9, 2025, the second wave from April 28 to May 4, 2025, and the third wave from June 23 to June 29, 2025. Each wave remained open for 1 week. The wave start dates were 56 days apart. This interval was selected for both theoretical and practical reasons. Methodological work on longitudinal panel designs emphasizes that time lags should be chosen according to the expected timing of the underlying psychological and behavioral processes, rather than solely by convention or convenience (Dormann and Griffin, 2015). In the present study, ESE was expected to fluctuate as students encountered barriers and evaluated their ability to continue exercising over several weeks. Persistence, absorption, value, and autonomy within EEI may develop through repeated participation, while PPF may change as students accumulate experiences relevant to endurance, strength, and everyday physical functioning. An eight-week interval was therefore considered theoretically reasonable for observing short-term accumulation and self-evaluative change. These processes may nevertheless operate at different rates, and the selected interval does not imply a fixed time course of physiological adaptation. The timing also allowed data collection to be completed before the main summer vacation period at the participating universities. In addition, the three-wave structure allowed the hypothesized temporally ordered pathway from Time 1 to Time 3 through Time 2 to be examined, which is preferable to cross-sectional mediation for evaluating longitudinal process models (Cole and Maxwell, 2003; Maxwell and Cole, 2007).

Each assessment referred to experiences during the previous 4 weeks, so the eight-week wave spacing and the four-week reporting window represented distinct design features. The reporting windows did not continuously cover the intervening semester. The design used a single nominal lag and could not evaluate sensitivity to shorter or longer intervals.

Questionnaires were administered offline using paper-and-pencil surveys. Trained research assistants distributed the questionnaires in classrooms, study rooms, or other quiet campus settings during the scheduled assessment periods. Before administration, the research assistants provided standardized instructions emphasizing that participation was voluntary, that there were no right or wrong answers, and that participants should answer according to their actual extracurricular exercise experiences during the previous 4 weeks. The questionnaires were completed independently and returned on site. The average completion time was approximately 8 min. After completing each wave, participants received a small non-cash gift as appreciation for their time.

To match participants’ responses across the three waves while reducing the use of directly identifying information in the analytic dataset, participants created a self-generated matching code at each wave. The code consisted of the participant’s university name, initials of their Chinese name, and the last four digits of their mobile phone number. The same coding rule was used at T1, T2, and T3, allowing the research team to link repeated questionnaires from the same participant across waves. The matching code was used only for longitudinal data linkage and data verification. After the three-wave dataset had been matched and checked, the matching code was removed from the analytic dataset before statistical analysis. The analytic study identifiers were unique. Participant-level verification confirmed unique linkage of the repeated questionnaires, with no unresolved duplicate-code or matching ambiguities. Original matching codes are not publicly disclosed because they contain potentially identifying information.

Several procedures were used to improve data quality. All questionnaires included an eligibility confirmation item indicating that the participant was at least 18 years old and had read and agreed to the informed consent statement. An instructed-response attention-check item was embedded in the questionnaire. The attention-check item was: “This item is used to check whether you are reading carefully; please select ‘Agree’ for this statement.” Questionnaires were screened for excessive missingness, patterned responding, and implausible response behavior. A questionnaire was treated as invalid if the participant failed the eligibility or consent item, failed the attention check, selected the same response option for nearly all items, provided logically inconsistent demographic information, or showed other clear evidence of careless responding. These procedures are consistent with recommendations that survey studies combine instructed-response items with response-pattern checks to identify careless or invalid responses (Curran, 2016; Meade and Craig, 2012). For scale scoring, core scale scores were prorated when at least 80% of the items within a scale were valid; otherwise, only that scale score for that wave was coded as missing. This threshold was applied separately to each scale (at least 8/10 ESE items, 16/20 EEI items, and 4/5 PPF items). Participants with a missing scale score could contribute their other observed scores to the longitudinal model.

At baseline, 1,900 questionnaires were distributed and 1,796 were returned, yielding a response rate of 94.53%. After applying the eligibility and data-quality screening criteria, 1,673 valid baseline questionnaires were retained, corresponding to 93.15% of returned questionnaires and 88.05% of distributed questionnaires. Among the 1,673 baseline participants, 1,543 provided valid follow-up data at Time 2, corresponding to a retention rate of 92.23% relative to the baseline analytic sample, and 1,455 provided valid follow-up data at Time 3, corresponding to an overall retention rate of 86.97%. Among T2 respondents, 1,387 also participated at T3 (89.89%); a further 68 participants returned at T3 after missing T2. Because longitudinal missingness is common in multiwave panel studies, the random-intercept cross-lagged panel models used full-information maximum likelihood estimation to retain all available information from participants with incomplete follow-up data. Full-information maximum likelihood is widely recommended for structural equation models with incomplete data under appropriate missing-data assumptions because it uses all available observed information rather than deleting cases listwise (Enders, 2022).

Sample size adequacy was considered in relation to the planned longitudinal structural equation model. Required sample size in structural equation modeling depends on model complexity, expected effect sizes, reliability of measures, distributional properties, and missing-data patterns; therefore, fixed rules of thumb are discouraged, and model-specific considerations are recommended (Muthén and Muthén, 2002; Wolf et al., 2013). The planned manifest-variable random-intercept cross-lagged panel model included three repeatedly measured constructs across three waves, random intercepts, autoregressive paths, cross-lagged paths, and within-wave correlations. Given the expectation of small-to-moderate within-person lagged effects and anticipated attrition across three waves, the recruitment plan aimed to distribute approximately 1,900 questionnaires at baseline to obtain at least 1,500 valid baseline cases and to retain more than 1,200 participants by the final wave. The achieved baseline analytic sample of 1,673 participants and the final-wave retention of 1,455 participants exceeded these minimum targets and supported estimation of the planned RI-CLPM, although precision for small indirect associations remained limited (Kline, 2023; Mulder, 2023).

### 2.2 Measurements

At each of the three waves, participants completed the same set of self-report measures. All items were administered in Chinese. To ensure consistency with the purpose of the present study, participants were instructed to answer all exercise-related items with reference to their extracurricular physical exercise during the past 4 weeks. Extracurricular physical exercise was defined as voluntary physical exercise or sport activity performed outside compulsory physical education classes, varsity-team training, or professional sport training. For the random-intercept cross-lagged panel model, total mean scores were calculated for each construct at each wave and used as manifest variables.

#### 2.2.1 Exercise self-efficacy

Exercise Self-Efficacy (ESE) was assessed using the Physical Activity Self-Efficacy Scale for College Students developed by Jiang et al. (2018). The scale measures students’ confidence in maintaining physical exercise when facing internal or external barriers. It contains 10 items and two dimensions: situational motivation, consisting of six items, and subjective support, consisting of four items. A representative item is: “Even when the weather is very bad, I will continue exercising.” Participants rated each item on a 5-point Likert scale ranging from 1 = strongly disagree to 5 = strongly agree. The mean of the 10 items was used as the ESE score, with higher scores indicating stronger exercise self-efficacy. The original validation study reported satisfactory reliability and validity among Chinese college students (Jiang et al., 2018).

#### 2.2.2 Extracurricular exercise involvement

Extracurricular Exercise Involvement (EEI) was measured using the Physical Exercise Involvement Scale for College Students developed by Dong (2017). The scale conceptualizes exercise involvement as an active, persistent, autonomous, and immersive psychological state during physical exercise. The original scale contains 20 items and four dimensions: vigor-persistence, consisting of six items; absorption and satisfaction, consisting of six items; value cognition, consisting of five items; and participation autonomy, consisting of three items. A representative item is: “When exercising, I am immersed in it.” Participants responded on a 5-point Likert scale ranging from 1 = completely inconsistent to 5 = completely consistent. In the present study, the item stem was adapted to emphasize extracurricular exercise, but the response format and scoring procedure were retained. The mean of the 20 items was used as the EEI score, with higher scores indicating greater extracurricular exercise involvement. The composite reflects psychological engagement rather than a direct measure of physical activity volume. Dong (2017) reported good internal consistency, split-half reliability, test–retest reliability, and factorial validity for the scale in Chinese university students.

#### 2.2.3 Perceived physical fitness

Perceived Physical Fitness (PPF) was assessed using the International Fitness Scale (IFIS), originally developed and validated by Ortega et al. (2011). The IFIS is a brief self-report measure of perceived physical fitness and includes five single-item domains: general fitness, cardiorespiratory fitness, muscular strength, speed/agility, and flexibility. A representative item is: “My general physical fitness is…” Participants rated each domain on a 5-point scale ranging from 1 = very poor to 5 = very good. The mean of the available items was used as a domain-balanced PPF composite when at least four domains were rated, with higher scores indicating better perceived physical fitness. Equal weighting preserves the breadth of the five domain ratings. A single-factor CFA was used as a diagnostic of their shared variance; the composite does not assume that the domains are interchangeable or provide an objective fitness assessment. The IFIS has shown evidence of validity and reliability in youth populations (Ortega et al., 2011), and the Chinese version has demonstrated moderate test–retest reliability among Chinese children and adolescents (Bao et al., 2022). Given that the present sample consisted of undergraduates, the reliability and longitudinal stability of the PPF scores were re-examined in the current study.

#### 2.2.4 Demographic covariates

Five demographic covariates were assessed at baseline: gender, academic year, major group, subjective socioeconomic status, and body mass index category. Gender was coded as male or female. Academic year was coded from Year 1 to Year 4. Major group was classified into science, technology, engineering, and mathematics; humanities and social sciences; medical and health-related majors; and arts or design-related majors. Subjective socioeconomic status was assessed by asking participants to rate their family socioeconomic position relative to other students. Body mass index was calculated from self-reported height and weight and then categorized for descriptive and sensitivity analyses. Only the BMI categories were available in the analytic records; continuous age, height, weight, BMI, university identifiers, and course arrangements were unavailable for the attrition and sensitivity analyses. Categorical covariates were represented by 11 dummy variables, with male gender, Year 1, STEM, low subjective socioeconomic status, and underweight as the reference categories.

### 2.3 Data analysis

Longitudinal models and CFAs were estimated using Mplus 8.3 (Muthén and Muthén, 1998–2017). Descriptive, reliability, missingness, and logistic regression analyses used R 4.6.1 and IBM SPSS Statistics 32 (build 134). Prior to the main longitudinal analyses, data were screened according to the predefined validity criteria described above. Scale scores for Exercise Self-Efficacy (ESE), Extracurricular Exercise Involvement (EEI), and Perceived Physical Fitness (PPF) were computed as item mean scores at each wave, with higher scores indicating higher levels of the corresponding construct. Core scale scores were prorated when at least 80% of the items within a scale were valid; otherwise, the scale score for that wave was treated as missing.

Descriptive statistics were calculated for all core variables at T1, T2, and T3, including means, standard deviations, skewness, and kurtosis. Internal consistency was evaluated using Cronbach’s alpha on item-complete cases separately for each scale and wave. Factor-specific composite reliability (CR) and average variance extracted (AVE) were calculated from standardized CFA loadings and residual variances as supplementary measurement-quality indicators (Cronbach, 1951; Fornell and Larcker, 1981). Cronbach’s alpha was interpreted as an index of internal consistency rather than as evidence of unidimensionality by itself (Tavakol and Dennick, 2011). CR and AVE were used only as descriptive evidence of measurement quality and were not used to estimate the manifest-variable RI-CLPM. Descriptive single-measure intraclass correlations (ICCs) were calculated from separate univariate random-intercept models fitted by restricted maximum likelihood, with wave included as a fixed effect. Each ICC was the person-level variance divided by the sum of person-level and residual variances. These descriptive mixed-model ICCs were distinguished from the random-intercept and within-person variance estimates of the multivariate RI-CLPM.

Measurement models were evaluated at each wave using robust maximum likelihood (MLR), treating the five-category items as approximately continuous and retaining partial item responses through FIML. ESE was specified with two correlated factors, EEI with four correlated factors, and PPF with a single diagnostic factor. Three-wave longitudinal CFA models then tested configural, metric, and scalar invariance by successively constraining corresponding loadings and intercepts. Residuals of the same item were allowed to correlate across waves. Partial scalar invariance was examined through limited relaxation of intercept constraints when full scalar invariance was not supported; all loadings remained constrained in the final partial model.

Measurement invariance was evaluated using changes in approximate fit: a decrease in CFI no greater than 0.010 and an increase in RMSEA no greater than 0.015, considered alongside increases in SRMR no greater than 0.030 for metric and 0.010 for scalar constraints (Chen, 2007). MLR-scaled difference tests were also reported, with decisions reflecting the combined evidence rather than the chi-square test alone. These analyses assess cross-wave comparability of the measures, while the primary RI-CLPM continued to use the observed scale means.

Missing longitudinal data were handled using full-information maximum likelihood (FIML). FIML uses all available observed information in model estimation and is generally preferable to listwise deletion under appropriate missing-data assumptions (Enders, 2022; Schafer and Graham, 2002). Participants with incomplete follow-up data were retained in the longitudinal models. FIML assumes missing at random (MAR) conditional on the observed variables in the model; MAR cannot be verified directly from the observed data. All significance tests were two-tailed, with *p* < 0.05 used as the conventional threshold for statistical significance (Graham, 2009).

Attrition analyses compared participants observed at all three waves with those missing at least one follow-up. Baseline scale scores were compared using Welch’s t tests and Hedges’ g with 95% confidence intervals; categorical characteristics were examined with Pearson chi-square tests and Cramér’s V. A multivariable logistic regression modeled complete three-wave participation (1 = complete, 0 = any subsequent wave missing) using standardized baseline ESE, EEI, and PPF scores and the 11 demographic dummy variables. Odds ratios for the scale scores therefore refer to a one-standard-deviation increment. Holm adjustment was applied separately to the three continuous comparisons, five categorical comparisons, and 14 regression coefficients (Holm, 1979).

To evaluate the longitudinal structure of the data, a series of competing models was estimated. A conventional cross-lagged panel model (CLPM) with freely estimated lagged paths was fitted as a comparison model. A free-lag random-intercept cross-lagged panel model (RI-CLPM) was estimated. The RI-CLPM separates stable between-person differences from within-person fluctuations by decomposing each repeated measure into a trait-like random intercept and a time-specific within-person component (Hamaker et al., 2015). In this model, autoregressive and cross-lagged paths are estimated among the within-person components, allowing the paths to be interpreted as whether deviations from an individual’s own usual level of one construct predict later deviations from that individual’s own usual level of another construct. An equal-lag RI-CLPM was estimated by constraining corresponding lagged paths from T1 to T2 and from T2 to T3 to be equal. This model tested whether the dynamic associations could be assumed to be stable across adjacent intervals. A covariate-sensitivity RI-CLPM included the 11 baseline demographic dummy variables as predictors of both the three random intercepts and the three T1 within-person components. A further model restricted covariate associations to the random intercepts. These models examined the stability of the within-person paths after accounting for demographic differences (Mulder, 2023; Orth et al., 2021; Usami, 2021; Zheng and Valente, 2023).

The structural models used MLR with FIML. Observed scale means entered the models without external person-mean centering. Random-intercept loadings and single-indicator within-person loadings were fixed to one, and observed residual variances were fixed to zero. Random intercepts were allowed to correlate and were orthogonal to all within-person components. Wave-specific observed means were freely estimated. The three T1 within-person components covaried, as did the innovation residuals at T2 and T3. All nine autoregressive and cross-lagged paths were estimated at each interval in the free-lag model.

Model fit was evaluated using multiple indices: chi-square test of model fit, root mean square error of approximation (RMSEA), comparative fit index (CFI), Tucker–Lewis index (TLI), standardized root mean square residual (SRMR), Akaike information criterion (AIC), Bayesian information criterion (BIC), and sample-size adjusted BIC. Because the chi-square test is sensitive to sample size, model evaluation emphasized the joint interpretation of approximate fit indices and information criteria. In general, CFI and TLI values of 0.90 or above were considered acceptable and values of 0.95 or above were considered good, whereas RMSEA and SRMR values of 0.08 or below were considered acceptable and values around 0.06 or below were considered good (Hu and Bentler, 1999). AIC, BIC, and sample-size adjusted BIC were used for relative model comparison, with lower values indicating better relative fit after accounting for model complexity (Akaike, 1974; Schwarz, 1978). Nested MLR models were compared using scaled likelihood-ratio difference tests based on their log-likelihoods, parameter counts, and scaling correction factors. Information criteria were interpreted only for models fitted to the same variables and likelihood basis. Final model selection was based on overall model fit, theoretical interpretability, parsimony, and whether equality constraints substantially worsened model fit (Kline, 2023).

After the final model was selected, standardized autoregressive and cross-lagged effects were interpreted at the within-person level. Autoregressive paths indicated whether higher-than-usual levels of a construct predicted higher-than-usual levels of the same construct at the next wave. Cross-lagged paths indicated whether higher-than-usual levels of one construct predicted later higher-than-usual levels of another construct after separating stable between-person differences. Random-intercept correlations were interpreted as between-person associations among students’ stable average levels of ESE, EEI, and PPF across the study period. Same-wave correlations at T1 and within-wave residual correlations at T2 and T3 were estimated in the full RI-CLPM but omitted from the main figures for visual clarity.

Between-interval differences were tested on the nine pairs of corresponding unstandardized lagged coefficients. A joint Wald test evaluated all nine equality restrictions, and individual differences were defined as the T2–T3 coefficient minus the T1–T2 coefficient. Individual tests used the covariance between the two estimates, and Holm-adjusted *p* values controlled multiplicity across the nine comparisons (Holm, 1979). Standardized coefficients were retained for describing effect magnitude. Random-intercept variances, their standard errors and 95% intervals, and the within-person variances and innovation variances were reported separately.

The hypothesized longitudinal indirect pathway was tested as the product of the within-person path from ESE_T1 to EEI_T2 and the within-person path from EEI_T2 to PPF_T3. The unstandardized product was estimated using MODEL CONSTRAINT, and the corresponding specific standardized indirect association was obtained using MODEL INDIRECT and STDYX standardization in Mplus. Because indirect effects are products of coefficients and their sampling distributions are often asymmetric, bias-corrected bootstrap confidence intervals were used as the primary supplementary inference for the indirect pathway (MacKinnon et al., 2004; Preacher and Hayes, 2008). Specifically, 2,000 participant-level bootstrap draws were used to obtain 95 and 99% bias-corrected confidence intervals for both unstandardized and standardized indirect associations. The bootstrap companion model used ML with the same data and structural specification; MLR-based standard errors and normal-theory Wald tests were reported alongside these intervals. Inference considered effect size, interval width, and the agreement between the Wald and bootstrap results, including whether the respective confidence intervals contained zero (Hayes and Scharkow, 2013; MacKinnon, 2008).

Sensitivity analyses also included participants with all nine composite scores observed and an attrition-informed model in which the female indicator predicted the random intercepts and T1 within-person components; baseline ESE was already part of the structural model. This analysis quantified sensitivity to observed attrition correlates under conditional covariate adjustment. A separate model added the within-person ESE_T1 → PPF_T3 path while retaining the adjacent-wave structure. This 16-week path links the same predictor and outcome occasions as the specific three-wave indirect association. Sensitivity was assessed through changes in the lagged coefficients, their intervals, and the indirect association.

Finally, all standardized cross-lagged effects from the final free-lag RI-CLPM were visualized using a forest plot. The forest plot was based on STDYX standardized estimates and approximate 95% Wald intervals computed from standardized estimates and their standard errors. This figure was used to complement the path diagram by showing both significant and nonsignificant cross-lagged effects in a single display.

## 3 Results

### 3.1 Participant flow and baseline characteristics

Figure 2 presents the participant flow across the three waves. The baseline analytic sample included 1,673 undergraduate students. Among these participants, 1,543 provided valid follow-up data at T2, and 1,455 provided valid follow-up data at T3. Thus, the retention rate was 92.23% from T1 to T2 and 86.97% from T1 to T3. A total of 1,387 students participated at all three waves (82.91%), giving a T2-to-T3 retention rate of 89.89% among T2 respondents. The remaining patterns comprised 156 students observed at T1 and T2 only, 68 observed at T1 and T3 only (4.06% intermittent missingness), and 62 observed only at T1. All nine composite scores were available for 1,383 students. One participant at T1 and two at each follow-up answered only three of the five PPF items, resulting in PPF score sample sizes of 1,672, 1,541, and 1,453. Their available ESE and EEI scores were retained through FIML. The analytic strategy allowed participants with incomplete longitudinal observations to contribute their available information.

**Figure 2:**
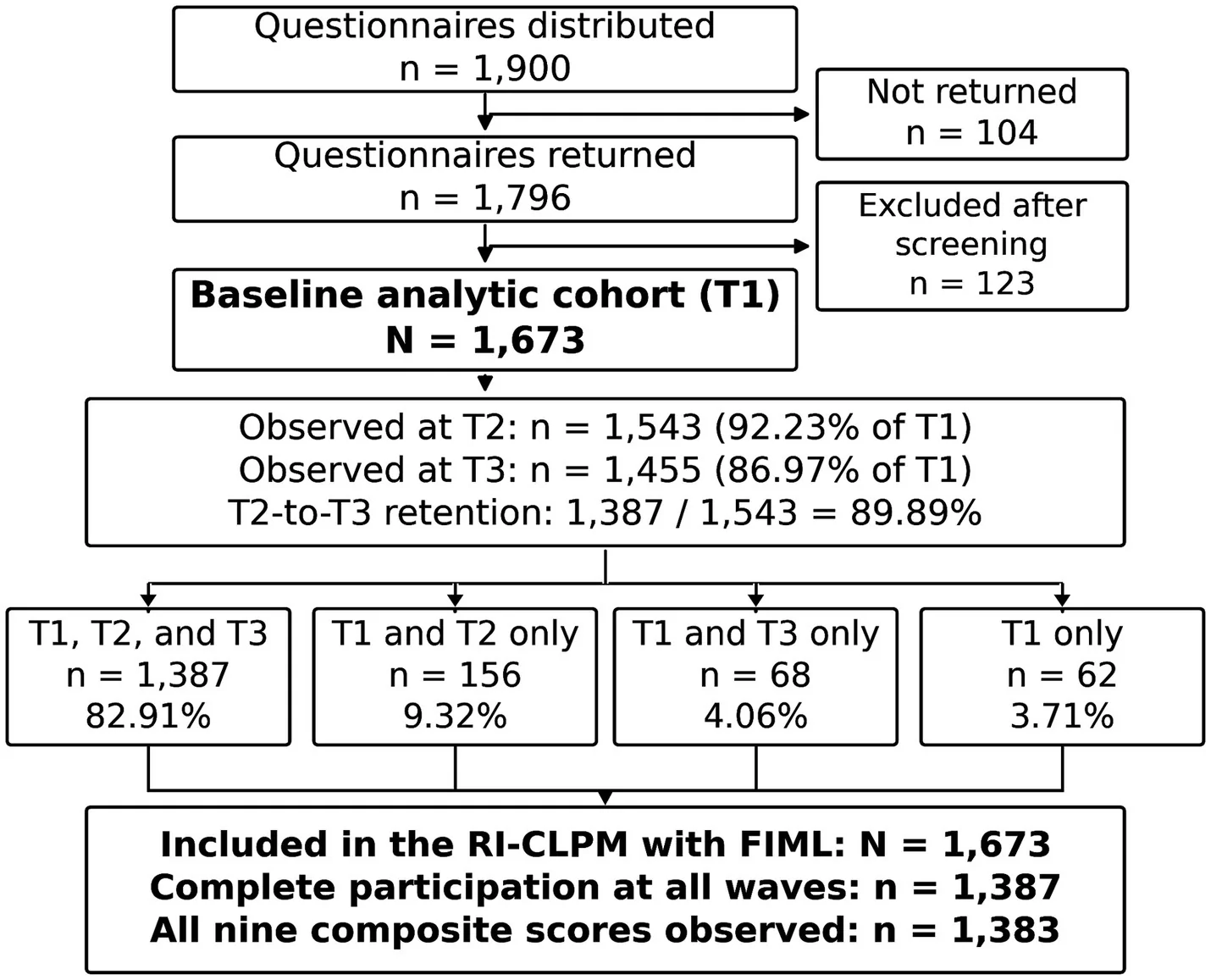
Participant flow and analytic sample across the three waves. T1, T2, and T3 denote the three measurement occasions. Follow-up patterns refer to participation, not complete item response. Of 1,455 T3 participants, 1,387 also participated at T2 and 68 returned after missing T2. T2-to-T3 retention is 1,387/1,543 = 89.89%. PPF score availability was 1,672 at T1, 1,541 at T2, and 1,453 at T3 because the 80% scoring threshold was applied separately to each scale. All nine composite scores were observed for 1,383 participants. FIML, full-information maximum likelihood; PPF, perceived physical fitness.

Baseline sample characteristics are shown in Table 1. The sample included slightly more female than male students and represented all four undergraduate academic years. The distribution of academic majors was broad, with science, technology, engineering, and mathematics forming the largest subgroup, followed by humanities and social sciences, medicine and health, and arts and design. Most participants reported middle subjective socioeconomic status, and most were classified as having normal weight based on BMI category. Overall, the baseline sample showed sufficient demographic heterogeneity for examining exercise-related psychological processes and perceived fitness among Chinese undergraduates.

**Table 1:** Baseline sample characteristics.

Variable	Category	*n*	%
Gender	Male	781	46.68
	Female	892	53.32
Academic year	Freshman	461	27.56
	Sophomore	438	26.18
	Junior	420	25.10
	Senior	354	21.16
Major group	STEM	655	39.15
	Humanities and social sciences	547	32.70
	Medicine and health	293	17.51
	Arts and design	178	10.64
Subjective socioeconomic status	Low	339	20.26
	Middle	964	57.62
	High	370	22.12
BMI category	Underweight	231	13.81
	Normal weight	1,209	72.27
	Overweight/obesity	233	13.93

*N* = 1,673. Percentages are based on the baseline analytic sample and may not sum to 100 because of rounding. BMI, body mass index; STEM, science, technology, engineering, and mathematics.

Baseline comparisons by follow-up participation are reported in Supplementary Table S1. Students with at least one missing follow-up had slightly higher baseline ESE, EEI, and PPF than three-wave participants (Hedges’ g = 0.275, 0.221, and 0.182, respectively). Female students were also more frequent in the incomplete-follow-up group. In the multivariable model (Supplementary Table S2; *N* = 1,672), higher baseline ESE was associated with lower odds of complete participation (OR per SD = 0.794, 95% CI [0.684, 0.922], Holm-adjusted *p* = 0.036), as was female gender (OR = 0.667, 95% CI [0.511, 0.870], Holm-adjusted *p* = 0.037). The model had modest explanatory and discriminatory performance (Nagelkerke R^2^ = 0.052; AUC = 0.634).

### 3.2 Measurement properties, descriptive statistics, and variance decomposition

The nine wave-specific CFAs showed good approximate fit (Supplementary Table S3): CFI ranged from 0.997 to 1.000, RMSEA from 0.000 to 0.027, and SRMR from 0.005 to 0.015. Standardized loading ranges were 0.585–0.801 for ESE, 0.605–0.837 for EEI, and 0.589–0.804 for PPF. Factor-specific reliability and extracted variance are reported in Supplementary Table S4. CR values were at least 0.777, whereas AVE values ranged from 0.411 to 0.682 and were below 0.50 for several factors. The ESE dimensions were strongly correlated across waves (*r* = 0.753–0.803), indicating substantial overlap. These results support reliable broad composites without establishing that all domains are sharply distinguishable.

Longitudinal measurement-model fit and nested comparisons are reported in Supplementary Tables S5, S6, respectively. EEI and PPF satisfied the approximate-fit-change criteria for full scalar invariance. ESE satisfied metric invariance, but full scalar constraints reduced fit. Partial scalar invariance was retained after freeing the intercept of subjective support Item 1 at T3 and situational motivation Item 6 at T1; all other intercept and loading constraints were retained. The final ESE model had CFI = 0.990, RMSEA = 0.016, and SRMR = 0.025, with changes relative to metric invariance of −0.009, 0.011, and 0.004, respectively. Scaled difference tests detected several departures from exact equality, so the invariance conclusions reflect the specified approximate-fit criteria.

Table 2 reports descriptive statistics, reliability estimates, and intraclass correlations for the core constructs. Across the three waves, mean scores for ESE, EEI, and PPF were all above the midpoint of the response scale, suggesting moderate-to-high levels of exercise self-efficacy, extracurricular exercise involvement, and perceived physical fitness. The mean scores showed a small downward trend over time, particularly for PPF, but the magnitude of change was modest. Skewness and kurtosis values did not indicate severe non-normality, supporting the use of manifest scale scores in subsequent longitudinal modeling.

**Table 2:** Descriptive statistics, reliability, and intraclass correlations for the core constructs.

Construct	Wave	*n*	M	SD	α	ICC	Skewness	Excess kurtosis
ESE	T1	1,673	3.416	0.872	0.882	0.519	−0.333	−0.491
	T2	1,543	3.389	0.872	0.878		−0.219	−0.684
	T3	1,455	3.377	0.890	0.888		−0.222	−0.704
EEI	T1	1,673	3.453	0.809	0.928	0.545	−0.299	−0.516
	T2	1,543	3.398	0.803	0.924		−0.229	−0.667
	T3	1,455	3.396	0.795	0.922		−0.343	−0.389
PPF	T1	1,672	3.491	0.894	0.777	0.559	−0.385	−0.465
	T2	1,541	3.466	0.930	0.791		−0.341	−0.590
	T3	1,453	3.409	0.961	0.812		−0.306	−0.689

ESE, exercise self-efficacy; EEI, extracurricular exercise involvement; PPF, perceived physical fitness; CI, confidence interval. α, Cronbach’s alpha, computed on item-complete cases separately for each scale and wave; ICC, descriptive single-measure intraclass correlation. Scale scores are available-item means when at least 80% of items are observed. ICCs are from separate univariate random-intercept models fitted with restricted maximum likelihood and fixed wave effects: ICC = person-level variance / (person-level variance + residual variance). These ICCs are distinct from the multivariate RI-CLPM variance decomposition in Supplementary Table S7.

The reliability estimates indicated internally consistent scores for the three constructs. Internal consistency was good for ESE, excellent for EEI, and acceptable for PPF. The descriptive single-measure ICCs from the univariate mixed models were 0.519 for ESE, 0.545 for EEI, and 0.559 for PPF. These values indicate that a substantial proportion of variance reflected stable between-person differences, while meaningful within-person variance remained available for longitudinal modeling. The multivariate RI-CLPM variance estimates are reported separately in Supplementary Table S7. Random-intercept variances were 0.332 for ESE (SE = 0.027, 95% CI [0.278, 0.386]), 0.266 for EEI (SE = 0.026, 95% CI [0.216, 0.316]), and 0.395 for PPF (SE = 0.031, 95% CI [0.335, 0.456]). Random-intercept and within-wave associations are reported in Supplementary Table S8. T2 and T3 innovation variances represent unexplained within-person variation conditional on preceding scores, rather than total within-person variances.

### 3.3 Model fit comparison and final model selection

Table 3 presents the fit indices for the competing longitudinal models. The conventional CLPM showed inadequate fit, particularly in terms of RMSEA and TLI, suggesting that a model without random intercepts did not adequately represent the longitudinal structure of the data. In contrast, the free-lag RI-CLPM showed excellent fit, χ^2^(3) = 6.667, *p* = 0.083, RMSEA = 0.027, CFI = 0.999, TLI = 0.991, SRMR = 0.008. This improvement indicates that separating stable between-person differences from within-person dynamics provided a better representation of the data.

**Table 3:** Model fit comparison.

Model	*k*	Scaled χ^2^ (df)	*p*	RMSEA [90% CI]	CFI	TLI	SRMR	AIC	BIC	SSA-BIC
CLPM, free lags	45	169.043 (9)	<0.001	0.103 [0.090, 0.117]	0.962	0.861	0.031	30878.547	31122.553	30979.595
RI-CLPM, free lags	51	6.667 (3)	0.083	0.027 [0.000, 0.055]	0.999	0.991	0.008	30721.807	30998.348	30836.328
RI-CLPM, equal lags	42	29.076 (12)	0.004	0.029 [0.016, 0.043]	0.996	0.989	0.018	30726.313	30954.053	30820.625
RI-CLPM, demographic adjustment	117	44.027 (36)	0.168	0.012 [0.000, 0.022]	0.998	0.994	0.010	30756.791	31391.209	31019.516

*N* = 1,673. *k* = number of free parameters; CLPM, cross-lagged panel model; RI-CLPM, random-intercept cross-lagged panel model; AIC, Akaike information criterion; BIC, Bayesian information criterion; SSA-BIC, sample-size adjusted BIC; RMSEA, root mean square error of approximation; CFI, comparative fit index; TLI, Tucker–Lewis index; SRMR, standardized root mean square residual. MLR-scaled statistics are reported. Equal-lag constraints versus the free-lag RI-CLPM yielded scaled Δχ^2^(9) = 22.425, *p* = 0.008. The demographic model includes 11 dummy predictors of both the three random intercepts and three T1 within-person components. Information criteria are not directly comparable across models with different covariate and likelihood specifications. The free-lag model was retained for primary interpretation; the lower BIC of the equal-lag model favors parsimony.

The equal-lag RI-CLPM also showed acceptable descriptive fit. However, constraining the lagged paths to be equal across the two adjacent intervals significantly worsened model fit relative to the free-lag RI-CLPM, scaled Δχ^2^(9) = 22.425, *p* = 0.008. The scaled comparisons of autoregressive, cross-lagged, and combined equality restrictions are reported in Supplementary Table S9. Although the equal-lag model was more parsimonious and had a lower BIC, the free-lag RI-CLPM was preferred because it provided better chi-square fit, lower AIC, and allowed the strength of lagged paths to vary between the early and later intervals. The lower BIC favored the more constrained model, indicating a trade-off between fit and parsimony. The covariate-sensitivity RI-CLPM also showed good descriptive fit; stability of its substantive paths was evaluated separately. Therefore, the free-lag RI-CLPM was retained as the final model for substantive interpretation.

### 3.4 Within-person autoregressive and cross-lagged effects

Table 4 presents the standardized lagged estimates from the final free-lag RI-CLPM, and Figure 3 visualizes the significant within-person lagged paths and the between-person random-intercept correlations. At the between-person level, the random intercepts of ESE, EEI, and PPF were positively correlated (ESE–EEI: *r* = 0.600; ESE–PPF: *r* = 0.618; EEI–PPF: *r* = 0.558; all *p* < 0.001), consistent with H1. Students who were generally higher in exercise self-efficacy across the study period also tended to be generally more involved in extracurricular exercise and to perceive themselves as physically fitter. This pattern indicates that stable between-person differences were substantial and theoretically meaningful.

**Table 4:** Standardized lagged effects from the final free-lag RI-CLPM.

Interval	Predictor	Outcome	Path type	*β*	*SE*	*z*	*p*	Approx. 95% CI
T1 → T2	ESE_T1	ESE_T2	Autoregressive	0.151	0.053	2.842	0.004	[0.047, 0.255]
T1 → T2	EEI_T1	ESE_T2	Cross-lagged	0.061	0.047	1.314	0.189	[−0.030, 0.153]
T1 → T2	PPF_T1	ESE_T2	Cross-lagged	0.026	0.051	0.514	0.607	[−0.074, 0.126]
T1 → T2	ESE_T1	EEI_T2	Cross-lagged	0.100	0.044	2.281	0.023	[0.014, 0.185]
T1 → T2	EEI_T1	EEI_T2	Autoregressive	0.280	0.047	5.991	<0.001	[0.189, 0.372]
T1 → T2	PPF_T1	EEI_T2	Cross-lagged	0.008	0.049	0.158	0.875	[−0.088, 0.103]
T1 → T2	ESE_T1	PPF_T2	Cross-lagged	0.059	0.043	1.376	0.169	[−0.025, 0.142]
T1 → T2	EEI_T1	PPF_T2	Cross-lagged	0.101	0.046	2.184	0.029	[0.010, 0.192]
T1 → T2	PPF_T1	PPF_T2	Autoregressive	0.217	0.053	4.107	<0.001	[0.113, 0.320]
T2 → T3	ESE_T2	ESE_T3	Autoregressive	0.190	0.049	3.851	<0.001	[0.093, 0.287]
T2 → T3	EEI_T2	ESE_T3	Cross-lagged	0.129	0.045	2.876	0.004	[0.041, 0.217]
T2 → T3	PPF_T2	ESE_T3	Cross-lagged	0.052	0.043	1.199	0.231	[−0.033, 0.136]
T2 → T3	ESE_T2	EEI_T3	Cross-lagged	0.179	0.044	4.085	<0.001	[0.093, 0.265]
T2 → T3	EEI_T2	EEI_T3	Autoregressive	0.191	0.053	3.580	<0.001	[0.086, 0.295]
T2 → T3	PPF_T2	EEI_T3	Cross-lagged	0.063	0.044	1.447	0.148	[−0.022, 0.149]
T2 → T3	ESE_T2	PPF_T3	Cross-lagged	0.003	0.042	0.069	0.945	[−0.080, 0.086]
T2 → T3	EEI_T2	PPF_T3	Cross-lagged	0.205	0.046	4.434	<0.001	[0.114, 0.295]
T2 → T3	PPF_T2	PPF_T3	Autoregressive	0.176	0.051	3.479	<0.001	[0.077, 0.275]

ESE, exercise self-efficacy; EEI, extracurricular exercise involvement; PPF, perceived physical fitness; CI, confidence interval. Estimates are STDYX standardized within-person parameters from the free-lag RI-CLPM (*N* = 1,673). SE, z, and *p* use MLR-based inference. Approximate 95% Wald intervals are calculated from unrounded standardized estimates and standard errors. Autoregressive paths describe within-person carryover; cross-lagged paths describe prospective associations between constructs after separating stable individual differences. These individual parameter tests do not test differences between the two intervals; the latter are reported in Supplementary Table S10.

**Figure 3:**
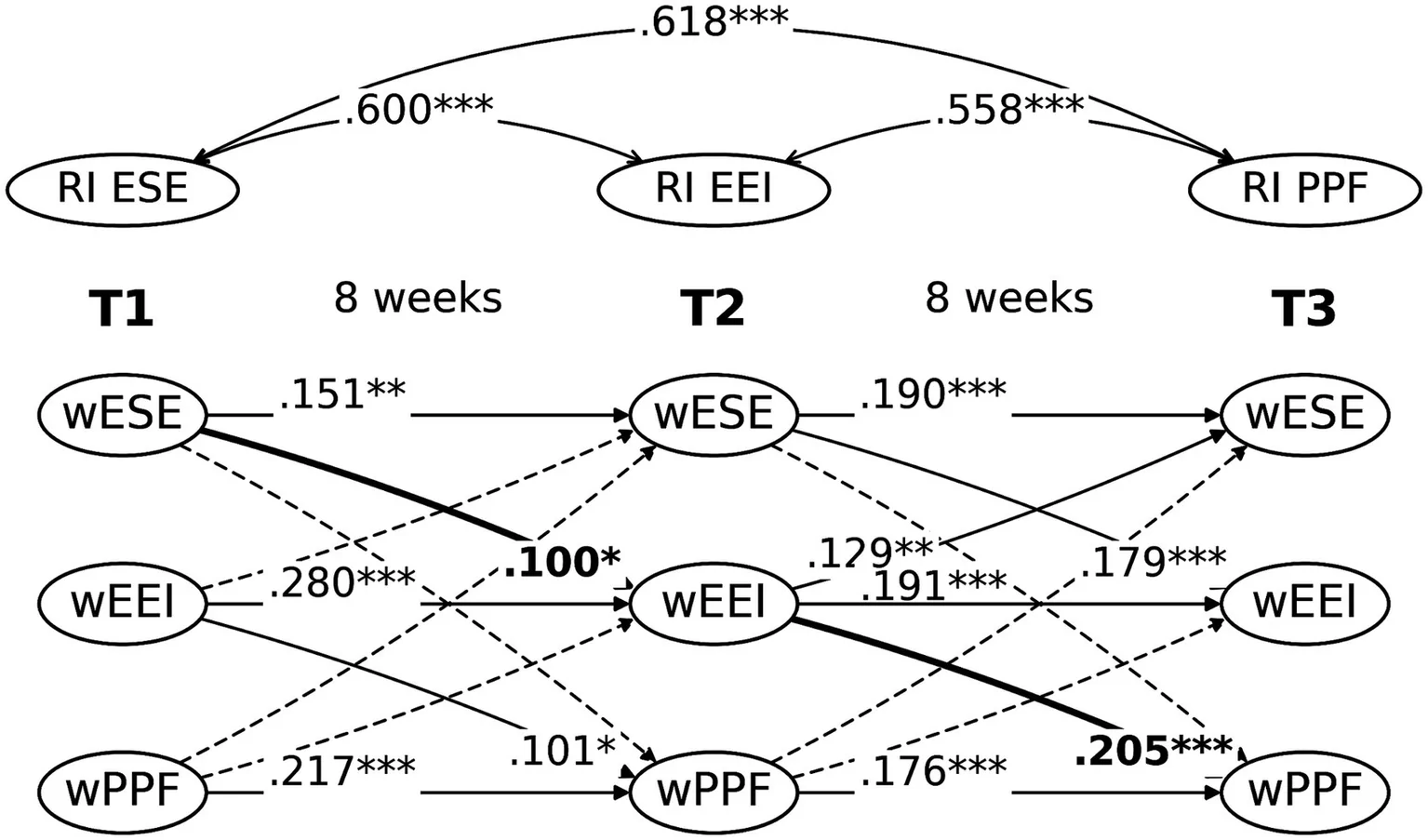
Standardized within-person and between-person estimates from the final free-lag RI-CLPM. ESE, exercise self-efficacy; EEI, extracurricular exercise involvement; PPF, perceived physical fitness; RI, random intercept; w, within-person component; T1–T3, measurement occasions. Values are STDYX standardized estimates. Solid arrows indicate lagged paths with *p* < 0.05; dashed arrows indicate nonsignificant lagged paths. The two paths defining the focal three-wave indirect association are shown with thicker solid lines. Curved double-headed arrows indicate correlations among random intercepts. Same-wave covariances and innovation covariances were estimated but are omitted for clarity. Significance symbols refer to the individual parameter tests, not between-interval comparisons. *p < 0.05, **p < 0.01, ***p < 0.001.

At the within-person level, all autoregressive paths were statistically significant. These effects indicate that when students reported higher-than-usual levels of ESE, EEI, or PPF at one wave, they tended to remain above their own usual levels on the same construct at the subsequent wave. However, the autoregressive coefficients were modest in magnitude, which is expected in RI-CLPM because stable trait-like differences are captured by the random intercepts rather than by within-person autoregressive paths.

The cross-lagged results revealed a clear dynamic pattern. From T1 to T2, higher-than-usual ESE predicted higher-than-usual EEI at the next wave, and higher-than-usual EEI predicted higher-than-usual PPF. From T2 to T3, the corresponding estimates were numerically larger: ESE again predicted subsequent EEI, and EEI again predicted subsequent PPF. In addition, EEI at T2 positively predicted ESE at T3, providing interval-specific evidence of a prospective feedback association. In contrast, PPF did not significantly predict later ESE or EEI, and the ESE-to-PPF paths were nonsignificant at both intervals. The repeated ESE-to-EEI and EEI-to-PPF associations were consistent with H2 and H3. Feedback paths examined under H5 showed evidence for EEI_T2 → ESE_T3, while the intervals for the PPF feedback paths remained compatible with small positive or negative associations.

The joint test of equality across all nine lagged paths was significant, Wald χ^2^(9) = 23.372, *p* = 0.005. Individual comparisons are presented in Supplementary Table S10. No individual path difference remained significant after Holm adjustment (minimum adjusted *p* = 0.234). The joint result therefore indicated nonuniformity in the overall lagged structure without establishing an adjusted significant difference for any specific path, including the EEI-to-ESE feedback association.

### 3.5 Core hypothesized pathway and longitudinal indirect effect

The specific three-wave indirect association is summarized in Table 5, which integrates Wald and bootstrap inference. The first component of the pathway was supported: higher-than-usual ESE at T1 predicted higher-than-usual EEI at T2, β = 0.100, *p* = 0.023. The second component was also supported: higher-than-usual EEI at T2 predicted higher-than-usual PPF at T3, β = 0.205, *p* < 0.001. These two adjacent-wave coefficients form the product for ESE_T1 → EEI_T2 → PPF_T3. The adjacent-wave ESE-to-PPF coefficients were nonsignificant, but they refer to different predictor occasions and do not establish complete mediation of the T1-to-T3 association.

**Table 5:** Specific three-wave indirect association from ESE at T1 to PPF at T3 through EEI at T2.

Scale	Estimate	MLR SE	Wald z	Wald *p*	95% Wald CI	95% BC bootstrap CI	99% BC bootstrap CI
Unstandardized b	0.02250	0.01172	1.920	0.055	[−0.00047, 0.04547]	[0.004, 0.051]	[−0.001, 0.062]
STDYX standardized	0.02038	0.011	1.902	0.057	[−0.001, 0.041]	[0.003, 0.045]	[−0.001, 0.056]

The specific pathway is within-person ESE_T1 → EEI_T2 → PPF_T3. ESE, exercise self-efficacy; EEI, extracurricular exercise involvement; PPF, perceived physical fitness; CI, confidence interval; MLR, robust maximum likelihood; BC, bias-corrected. Standard errors, z tests, *p* values, and Wald intervals are from the MLR model. The companion ML model used the same data and structural specification with 2,000 completed participant-level bootstrap resamples; its intervals are bias-corrected, not BCa intervals. The standardized association was estimated through MODEL INDIRECT with STDYX standardization. The unstandardized and standardized Wald intervals include zero, whereas the corresponding 95% BC bootstrap intervals exclude zero.

The three-wave indirect effect from ESE_T1 to PPF_T3 through EEI_T2 was small and positive, b = 0.02250, SE = 0.01172; the standardized estimate was 0.02038. The normal-theory Wald test was marginal and did not reach the conventional 0.05 threshold, z = 1.920, *p* = 0.055. However, the bias-corrected bootstrap analysis with 2,000 draws indicated that the 95% confidence interval for the indirect effect did not include zero, 95% CI [0.004, 0.051], although the 99% confidence interval slightly included zero, 99% CI [−0.001, 0.062]. The standardized 95% bias-corrected interval was [0.003, 0.045]. Together, these results provided qualified support for H4: the estimated association was small and its statistical interpretation depended on the inferential method.

Taken together, the results provide qualified support for the proposed within-person dynamic pathway. Higher-than-usual exercise self-efficacy predicted later higher-than-usual extracurricular exercise involvement, and higher-than-usual extracurricular exercise involvement predicted later higher-than-usual perceived physical fitness. The indirect pathway was supported by the 95% bootstrap confidence interval, whereas the normal-theory Wald test provided only marginal evidence. The finding is therefore a small specific longitudinal indirect association requiring replication.

### 3.6 Overall pattern of cross-lagged effects

Figure 4 provides a forest plot of all standardized cross-lagged effects from the final free-lag RI-CLPM. This plot complements Figure 3 by displaying both significant and nonsignificant cross-lagged paths with their approximate 95% Wald intervals. The most consistent cross-lagged association was from ESE to subsequent EEI, which was positive at both intervals and numerically larger from T2 to T3. EEI also consistently predicted subsequent PPF at both intervals, with a numerically larger estimate in the later interval. The only significant reverse effect was from EEI at T2 to ESE at T3, indicating a prospective association with later exercise confidence during that interval. By contrast, the paths from PPF to later ESE or EEI were small and nonsignificant. The figure describes the direction, magnitude, and uncertainty of these associations; formal between-interval inference is given by the adjusted comparisons in Supplementary Table S10.

**Figure 4:**
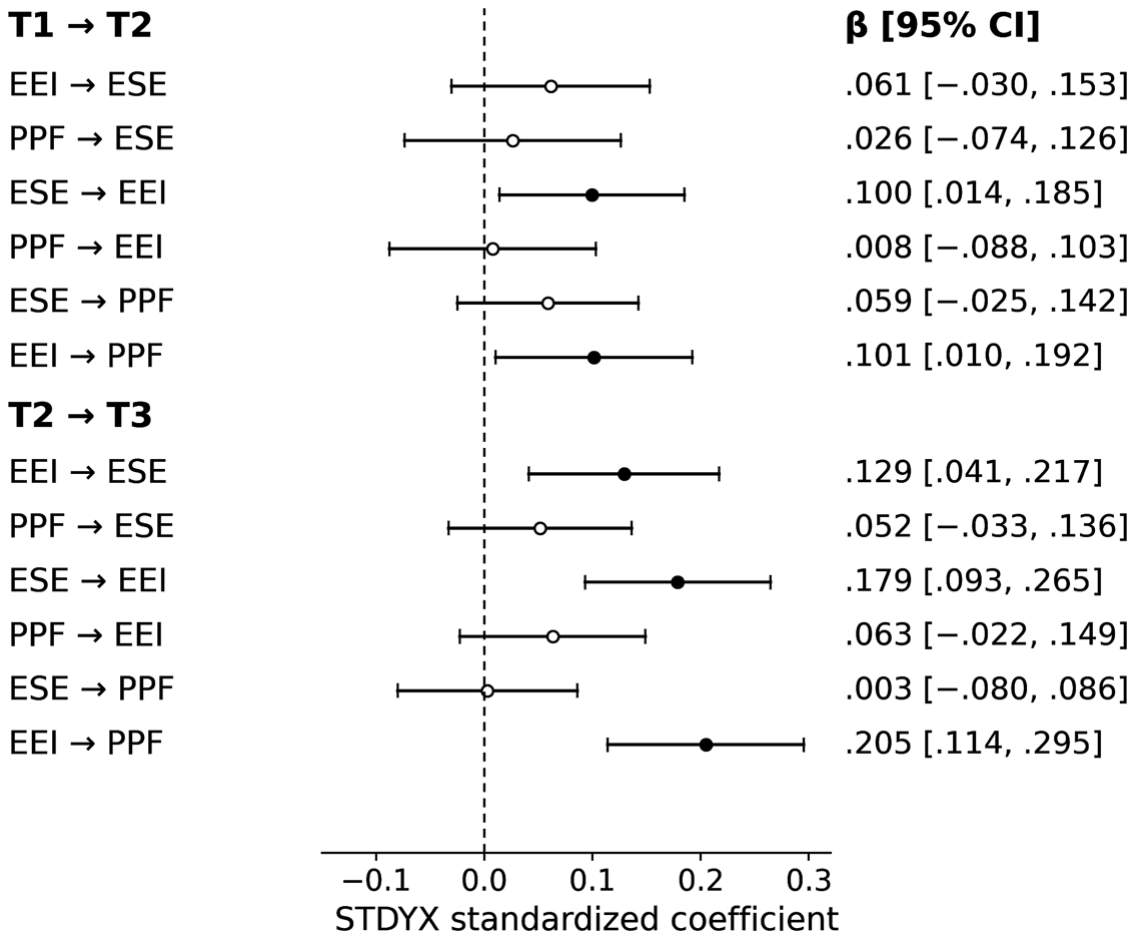
Forest plot of standardized cross-lagged effects from the final free-lag RI-CLPM. ESE, exercise self-efficacy; EEI, extracurricular exercise involvement; PPF, perceived physical fitness; T1–T3, measurement occasions. Points are STDYX estimates and horizontal bars are approximate 95% Wald confidence intervals based on unrounded standardized estimates and standard errors. The dashed vertical line marks zero. Positive coefficients indicate that higher-than-usual levels of the predictor were associated with higher-than-usual levels of the outcome at the next wave. The plot shows individual associations; interval differences were evaluated separately. Filled points indicate *p* < 0.05; open points indicate *p* ≥ 0.05.

### 3.7 Sensitivity analyses

Model fit and coefficient-stability summaries are reported in Supplementary Table S11. Adjusting for all baseline demographic categories as predictors of the random intercepts and T1 within-person components preserved the significance classification of all 18 lagged paths, with a maximum absolute change in β of 0.022 (Supplementary Table S12). The corresponding RI-only and attrition-informed female-adjusted models also retained all 18 significance classifications, with maximum absolute changes of 0.006 and 0.004, respectively (Supplementary Table S13). The nine-score complete-case model (*N* = 1,383) retained all path directions and significance classifications, with a maximum absolute change of 0.023. These comparisons indicate limited sensitivity of the principal lagged associations to the examined specifications, while the observed baseline differences by participation pattern remain relevant to missing-data interpretation.

In the model containing the additional 16-week within-person ESE_T1 → PPF_T3 path, that coefficient was small and nonsignificant (β = −0.032, 95% CI [−0.131, 0.068], *p* = 0.534; Supplementary Table S14). The specific indirect association remained positive and similar in magnitude across the sensitivity models, with unstandardized estimates of 0.019–0.023 and standardized estimates of 0.018–0.021 (Supplementary Table S15). Its unstandardized Wald *p* values ranged from 0.055 to 0.087. Thus, the sensitivity results preserved the small positive point estimate without resolving its inferential uncertainty.

## 4 Discussion

### 4.1 Principal interpretation of the findings

This study examined the dynamic associations among exercise self-efficacy, extracurricular exercise involvement, and perceived physical fitness in Chinese undergraduates using a three-wave free-lag RI-CLPM. The central contribution of the study is that it moved beyond the question of whether more confident or more involved students differ from less confident or less involved students. Instead, it examined whether fluctuations within the same student over time predicted later fluctuations in another construct. This distinction is important because stable between-person differences were substantial for all three constructs, meaning that the observed associations included substantial enduring differences among students as well as occasion-specific variation. After separating these stable differences, the within-person results supported the core theoretical sequence proposed in Figure 1: when students reported higher-than-usual exercise self-efficacy, they subsequently reported higher-than-usual extracurricular exercise involvement; when they reported higher-than-usual involvement, they subsequently reported higher-than-usual perceived physical fitness.

Exercise self-efficacy was prospectively associated with subsequent involvement, and involvement was associated with later perceived fitness. This ordering provides a useful hypothesis about the connection between capability beliefs and self-evaluation, although the small specific indirect association leaves the explanatory role of EEI provisional. This is consistent with the broader exercise psychology literature showing that self-efficacy is both a determinant and a consequence of physical activity behavior (McAuley and Blissmer, 2000). In the present study, self-efficacy showed both stable between-person variation and temporal within-person associations: within-person increases in exercise self-efficacy predicted later increases in exercise involvement. This pattern draws attention to sustained, autonomous, and personally valued exercise engagement as a plausible context in which capability beliefs acquire prospective relevance.

### 4.2 Extracurricular exercise involvement as the central dynamic process

A key implication of the findings is that extracurricular exercise involvement represents a motivational–experiential construct distinct from behavioral exposure or exercise frequency. In this study, involvement represented a higher-quality form of exercise engagement, including persistence, absorption, satisfaction, value cognition, and participation autonomy. The repeated paths from exercise self-efficacy to later extracurricular exercise involvement suggest that students who become more confident in their ability to exercise may be more capable of converting intention into active engagement. The anticipatory capability judgment represented by ESE may reduce perceived barriers to participation, while the experiential competence and autonomy emphasized by self-determination theory may support continued engagement. This interpretation is theoretically consistent with self-determination theory, which emphasizes that competence and autonomy are central to sustained motivation and self-regulated behavior (Deci and Ryan, 2000). It is also consistent with evidence that more autonomous forms of exercise motivation are generally associated with better exercise adherence and behavioral outcomes (Teixeira et al., 2012).

The repeated paths from extracurricular exercise involvement to later perceived physical fitness are consistent with an appraisal process in which the quality of participation precedes changes in perceived bodily capacity. Repeated, effortful, absorbing, and personally meaningful exercise experiences may provide occasions for students to notice and evaluate their bodily capacity. Such involvement may provide bodily feedback, mastery experiences, and affective reinforcement. Reviews of physical activity maintenance emphasize that sustained behavior depends on more than initial motivation; maintenance requires self-regulatory resources, personally meaningful motives, environmental support, and repeated enactment of the behavior (Kwasnicka et al., 2016). Similarly, affective responses to exercise are relevant to future motivation and behavior, suggesting that the experiential quality of exercise can shape whether students continue to engage (Rhodes and Kates, 2015). Although outside the exercise domain, related motivational research has also treated engagement as a mediating process through which motivational and contextual conditions are translated into downstream outcomes (Nusraningrum et al., 2024). The present associations place this proposed role of engagement in a specific exercise context. Direct measurement of mastery experiences, affect, and activity dose would be needed to distinguish these possible explanations of the EEI-to-PPF association.

### 4.3 Reciprocal dynamics between exercise self-efficacy and exercise involvement

The later interval revealed a significant reverse path from extracurricular exercise involvement at T2 to exercise self-efficacy at T3. This finding is theoretically meaningful because it is consistent with a feedback association from involvement to subsequent confidence. A possible account is that repeated participation provides more concrete evidence of successful performance, making capability judgments easier to update. Early fatigue or setbacks may convey less consistent mastery information when an exercise routine is still exploratory. This pattern fits well with the idea that mastery experiences are a major source of efficacy beliefs. When students persist in exercise, experience progress, and feel absorbed or satisfied during activity, they may become more confident in their future ability to continue exercising.

Importantly, this reciprocal pattern was not perfectly symmetrical across the whole study. The reverse effect from involvement to self-efficacy emerged from T2 to T3 but not from T1 to T2. The between-interval EEI-to-ESE difference was nonsignificant before and after Holm adjustment (unadjusted *p* = 0.173; adjusted *p* = 1.000), leaving the timing of feedback uncertain. One plausible interpretation is that involvement-related experiences require time to become psychologically consequential. Early in the semester, confidence may help students initiate or increase involvement; later, accumulated involvement may provide enough successful experience to strengthen confidence. This pattern also aligns with research on habit formation, which suggests that repeated behavior over time can gradually increase automaticity and reduce the effort required for continued action (Lally et al., 2010). The present study did not directly measure habit or automaticity, so this interpretation should be treated as theoretical rather than conclusive. The three-wave design also cannot directly identify thresholds or cumulative mastery effects. Repeated assessments of mastery attribution, coping with setbacks, and habit strength would help distinguish an accumulating feedback process from sampling variation or changing semester conditions.

### 4.4 Perceived physical fitness as a self-evaluation and potential feedback source

The results also clarify the role of perceived physical fitness. In the within-person model, perceived physical fitness did not significantly predict later exercise self-efficacy or extracurricular exercise involvement. In this sample and over the eight-week intervals, prospective associations were more consistently detected from involvement to PPF. However, the PPF feedback intervals included small positive associations, so the nonsignificant estimates leave its potential motivational role unresolved.

This finding is useful because perceived physical fitness can be theoretically ambiguous. On the one hand, feeling physically fit might motivate students to exercise more. On the other hand, perceived fitness may be a reflective appraisal that changes after students accumulate exercise experiences. The present results are consistent with this appraisal role, while the feedback role may depend on when and how bodily perceptions are interpreted. This pattern is consistent with research showing positive links between physical activity and physical self-concept, while also highlighting that self-perceptions may develop from repeated activity experiences rather than automatically driving future behavior (Babic et al., 2014). For university students, perceived physical fitness may therefore be considered a meaningful self-evaluative outcome alongside its potential feedback role: it captures how students interpret their own bodily capacity after engaging in exercise (Ma et al., 2024; Wang, 2019; Yang et al., 2024).

Several features may help explain the weak observed feedback. A five-domain mean can obscure differences between, for example, perceived endurance and flexibility, while a fixed eight-week lag may miss more immediate or longer-term responses to bodily perceptions. Moreover, PPF may vary with mood, body image, social comparison, or attribution of progress. A perceived improvement may strengthen confidence when it is attributed to personal effort, but have less impact when it is viewed as temporary or dependent on circumstances. The role of objectively measured fitness in subsequent engagement requires separate investigation.

This interpretation has practical importance. A plausible intervention hypothesis is that fitness-related feedback could be paired with achievable exercise goals, personally valued activities, and opportunities for continued mastery. Such combinations may help students interpret and use bodily information; their effectiveness requires experimental evaluation. Future evaluations could examine whether supportive interpretation of fitness information contributes to continued engagement (Brown et al., 2024; Huang et al., 2024; Yuan et al., 2024).

### 4.5 Interpretation of the longitudinal indirect effect

The hypothesized longitudinal indirect pathway from exercise self-efficacy at T1 to perceived physical fitness at T3 through extracurricular exercise involvement at T2 was small and positive. The normal-theory Wald test was marginal, whereas the bias-corrected bootstrap 95% confidence interval did not include zero. The standardized estimate of 0.02038 indicates a limited magnitude even though the 95% bootstrap interval excluded zero. The 99% interval included zero, and the sensitivity-model Wald tests remained near or above the conventional threshold. The result therefore provides tentative evidence consistent with a specific indirect association that warrants replication.

Indirect effects are products of two paths and often have asymmetric sampling distributions. Resampling approaches are commonly recommended for mediation because they do not rely as strongly on the assumption that the product term is normally distributed (Preacher and Hayes, 2008). At the same time, different inferential approaches can disagree, especially when effects are small or near a significance threshold (Hayes and Scharkow, 2013). The results provide qualified evidence for a small longitudinal indirect pathway. The direction of the estimate is consistent with the hypothesized sequence, while its uncertainty and the observational design limit mechanistic interpretation.

The RI-CLPM locates this association in deviations from each student’s typical level after separating stable between-person differences. The product therefore describes a specific temporal sequence within the broader reciprocal system, which also contains autoregressive and other cross-lagged routes from T1 to T3. Adding the long-lag ESE_T1 → PPF_T3 path left the focal product similar in magnitude; its nonsignificant coefficient does not establish complete mediation. For theory development, the useful implication is that involvement may be one prospective link between capability beliefs and self-evaluative fitness, with its size and persistence potentially contingent on repeated experience and context.

### 4.6 Theoretical implications

A theoretical implication is support for a process-oriented view of exercise engagement among university students. The findings suggest that exercise-related beliefs, involvement, and physical self-evaluation unfold dynamically over time. The conceptual distinction introduced earlier remains important: exercise behavior should not be reduced to frequency or duration alone. The associations connect the quality of involvement with both prior confidence and subsequent perceived fitness, while leaving its explanatory magnitude open to further testing.

A related theoretical implication concerns the role of self-efficacy. The findings suggest that exercise self-efficacy may participate in a dynamic feedback system involving the quality of exercise engagement. Self-efficacy predicted later involvement, and later involvement also predicted subsequent self-efficacy during the second interval. This pattern is compatible with a reciprocal mastery account, although mastery was not measured and the between-interval contrast was nonsignificant. The detectable feedback was specific: involvement fed back to self-efficacy, whereas perceived fitness did not significantly feed back to either self-efficacy or involvement. This specificity helps refine the model. The central reciprocal relation may be between confidence and involvement, whereas perceived fitness may be a downstream evaluative consequence over the time scale examined (Bao, 2025; Chen and Xiang, 2025; Gao et al., 2025; Sheng et al., 2025; Taylor et al., 2025; Zhou et al., 2025).

The methodological implication concerns the level of inference. The study illustrates why separating between-person and within-person processes is crucial in exercise psychology. Students who generally have higher exercise self-efficacy also tend to be more involved and perceive themselves as fitter. However, such between-person associations do not automatically imply that increases in self-efficacy within the same student will necessarily be followed by later increases in involvement or perceived fitness. By using an RI-CLPM, the present study provides a more precise account of intraindividual dynamics. This supports broader calls for longitudinal research to align theoretical models of change with temporal design and statistical modeling (Collins, 2006; Hopwood et al., 2022; Hamaker et al., 2015; Mulder and Hamaker, 2021; Orth et al., 2021; Usami, 2021).

### 4.7 Practical implications

The findings identify ESE and the quality of EEI as candidate targets for campus exercise-promotion research. These implications are preliminary and require experimental or quasi-experimental evaluation. Potential strategies include helping students experience manageable mastery, choose personally preferred activities, overcome common barriers, and receive feedback that reinforces progress. For example, future programs could evaluate progressive extracurricular exercise modules in which students set realistic goals, select preferred activity types, monitor effort and enjoyment, and receive structured encouragement from instructors or peers (Ashford et al., 2010; Bateman et al., 2022; Sheeran et al., 2016; Williams and French, 2011).

The prospective associations of extracurricular exercise involvement also encourage evaluation of participation quality alongside attendance-based indicators. Attendance and involvement capture different aspects of participation, and their respective contribution to sustained engagement remains an empirical question. The present findings point to the importance of autonomy, value, absorption, and persistence. Campus programs could evaluate diverse activities with flexible entry points, including noncompetitive fitness groups, recreational sport clubs, walking or running groups, strength-training workshops, and low-barrier activities for students with low confidence. Such designs offer opportunities to examine whether self-endorsed and personally meaningful experiences support continued participation (Bauman et al., 2012; Brown et al., 2024; Van Luchene and Delens, 2021).

The distinction between perceived fitness and objective performance is also important for intervention practice. Evidence from subjective fitness ratings has limited applicability to feedback based on objective fitness tests. A useful research question is whether pairing fitness feedback with actionable guidance supports engagement more effectively than providing feedback alone. For example, an intervention could provide feedback on perceived endurance, strength, flexibility, and speed/agility, followed by specific activity suggestions and short-term mastery goals. This approach may help students interpret bodily feedback as evidence of progress rather than as a fixed evaluation of ability (Babic et al., 2014; Ma et al., 2024; Ruiz et al., 2009).

The interval-specific feedback association also motivates research on how confidence and involvement change over repeated exercise experiences. The effective duration and format of exercise-support programs require comparative intervention research. Repeated practice over several weeks is more consistent with evidence on behavior maintenance and habit development (Kwasnicka et al., 2016; Lally et al., 2010). Comparisons of different program durations, accompanied by repeated measures of mastery and involvement, could clarify how exercise support can be integrated with the rhythm of the semester.

## 5 Limitations and future directions

Several limitations should be considered when interpreting the findings. Although the RI-CLPM strengthened the analysis by separating stable between-person differences from within-person dynamics, the study remained observational. Therefore, causal conclusions should be made cautiously. Unmeasured time-varying factors may have influenced the observed paths, including academic workload, examination stress, weather, access to sport facilities, injury, peer exercise climate, and changes in public physical education requirements. Future studies could strengthen causal inference by combining longitudinal panel designs with intervention or quasi-experimental components, such as campus-based exercise programs that manipulate self-efficacy support or involvement-enhancing activity structures. This would better align the statistical model with causal assumptions (Hernán and Robins, 2020; Little, 2013).

The study used three waves separated by approximately 8 weeks. This design was theoretically defensible for examining semester-level changes, but it cannot determine whether the same processes operate over shorter or longer time scales. Exercise self-efficacy may fluctuate from week to week or even from day to day, whereas perceived physical fitness may require longer periods of repeated exercise to change. Time-lag selection is a central issue in longitudinal modeling because different lags can produce different estimates of dynamic associations (Gollob and Reichardt, 1987). Three waves also provide limited leverage for distinguishing long-term change from short-term fluctuations or for identifying nonlinear, threshold, cumulative, or decaying processes. Future research should test whether the ESE–EEI–PPF pathway replicates across alternative time scales, such as weekly diary designs, monthly panels, four- or five-wave semester designs, or intensive longitudinal designs. With more assessment occasions, latent curve models with structured residuals, latent change score models, or continuous-time models could clarify the timing of these associations (Collins, 2006; Dormann and Griffin, 2015).

The study used manifest total mean scores in the RI-CLPM, so measurement error was not explicitly separated from within-person variation. Longitudinal CFAs supported full scalar invariance for EEI and PPF and partial scalar invariance for ESE under approximate-fit criteria. The two noninvariant ESE intercepts, overlap between ESE dimensions, and AVE values below 0.50 for several factors indicate that measurement precision and comparability remain imperfect. Future studies could estimate multiple-indicator RI-CLPMs after evaluating the measurement structure and cross-wave comparability. This would allow researchers to determine whether the within-person paths remain stable when measurement error is separated from true score variance. Such analyses would be particularly useful for perceived physical fitness, which was measured with a brief five-item scale (Kline, 2023; Little, 2013).

All core constructs were measured by self-report. Although the three-wave design reduced some concerns associated with purely cross-sectional self-report research, common method variance and subjective interpretation remain possible. In particular, perceived physical fitness is a subjective psychological construct. Recent mood, body image, self-esteem, social comparison, academic stress, and prior exercise experiences may influence these ratings without corresponding changes in physiological fitness. Future research should combine perceived fitness with objective or performance-based indicators, such as cardiorespiratory fitness tests, strength tests, flexibility tests, body composition, or official university physical fitness records. It would also be valuable to include objective or semi-objective exercise indicators, such as accelerometer data, exercise logs, or campus sport facility records. This would help determine whether extracurricular exercise involvement predicts both perceived and objectively measured fitness changes (Ortega et al., 2011; Ruiz et al., 2009; Wang, 2019).

The sample was drawn from eight universities in Sichuan Province, China. Although the multisite design improved sample heterogeneity, university identifiers were unavailable in the analytic data, preventing assessment of university-level clustering or site-specific sensitivity. Continuous age and BMI were also unavailable for further adjustment. Accordingly, the findings may not generalize to all Chinese undergraduates or to students in other cultural and institutional contexts. Universities differ in sport facilities, campus exercise culture, public physical education requirements, climate, and academic pressure. Future studies should replicate the model in other regions of China and in cross-cultural samples. It would also be valuable to test whether the pathways differ by gender, academic year, BMI category, baseline activity level, or participation in organized sport.

Selective attrition is another limitation. Incomplete-follow-up participants had slightly higher baseline scores, and baseline ESE and female gender were associated with lower completion odds. The attrition-informed and complete-case models yielded similar lagged estimates, but they do not establish MAR or eliminate bias from unobserved reasons for missingness. Self-generated codes combining university affiliation, name initials, and partial telephone numbers retain some potential for identification and code collisions. Randomly assigned study identifiers may offer a more privacy-preserving approach to future longitudinal follow-up.

The indirect effect should be interpreted with appropriate caution. The 95% bias-corrected bootstrap interval supported the indirect pathway, but the normal-theory Wald test was marginal and the 99% bootstrap interval slightly included zero. This pattern suggests a small and statistically modest effect rather than a strong mediation effect. Future studies should pre-register the mediation hypothesis, use larger or more intensive longitudinal samples, and consider complementary inference methods for indirect effects, such as Monte Carlo confidence intervals or distribution-of-the-product approaches. Such strategies would provide a clearer assessment of the magnitude and replicability of the specific indirect association linking exercise self-efficacy, involvement, and perceived fitness (Hayes and Scharkow, 2013; MacKinnon, 2008; MacKinnon et al., 2004).

## 6 Conclusion

This three-wave RI-CLPM study showed that, after separating stable between-person differences from within-person fluctuations, higher-than-usual exercise self-efficacy predicted later higher-than-usual extracurricular exercise involvement, and higher-than-usual involvement predicted later higher-than-usual perceived physical fitness among Chinese undergraduates. Extracurricular exercise involvement also predicted later exercise self-efficacy during the second interval, consistent with a possible feedback association, although adjusted between-interval differences were not significant. The longitudinal indirect pathway from exercise self-efficacy to perceived physical fitness through extracurricular exercise involvement was small and received tentative support from the 95% bias-corrected bootstrap interval, while the Wald test remained marginal. Overall, ESE and autonomous, meaningful extracurricular exercise involvement warrant evaluation as candidate intervention targets. The subjective PPF measure, regional university sample, and observational design limit generalization and causal interpretation, and the small indirect association requires replication.

## Data Availability

The original contributions presented in the study are included in the article/Supplementary material, further inquiries can be directed to the corresponding author.
