# Supplementary material for: Dynamic associations among exercise self-efficacy, extracurricular exercise involvement, and perceived physical fitness in Chinese undergraduates: a three-wave RI-CLPM study
Source: Front Psychol. 2026 Sep 29;17:1909123. doi: 10.3389/fpsyg.2026.1909123 (PMC13658085; doi:10.3389/fpsyg.2026.1909123)
Supplement: Supplementary file 1 [file Table_1.DOCX]

**Supplementary materials**

Exercise self-efficacy, extracurricular exercise involvement, and perceived physical fitness: a three-wave RI-CLPM study

**Table S1**

Baseline characteristics by three-wave participation status

| **Characteristic** | **Complete follow-up (n = 1,387)** | **Incomplete follow-up (n = 286)** | **Test** | **p** | **Holm p** | **Effect size [95% CI]** |
| --- | --- | --- | --- | --- | --- | --- |
| ESE at T1 | 3.375 (.877) | 3.613 (.818) | Welch t | < .001 | < .001 | g = .275 [.147, .402] |
| EEI at T1 | 3.422 (.813) | 3.601 (.772) | Welch t | < .001 | < .001 | g = .221 [.094, .349] |
| PPF at T1 | 3.463 (.901) | 3.626 (.843) | Welch t | .004 | .004 | g = .182 [.055, .309] |
| Gender |  |  | χ²(1) = 7.842 | .005 | .026 | V = .068 |
| Male | 669 (48.23%) | 112 (39.16%) |  |  |  |  |
| Female | 718 (51.77%) | 174 (60.84%) |  |  |  |  |
| Academic year |  |  | χ²(3) = 6.901 | .075 | .225 | V = .064 |
| Year 1 | 393 (28.33%) | 68 (23.78%) |  |  |  |  |
| Year 2 | 349 (25.16%) | 89 (31.12%) |  |  |  |  |
| Year 3 | 357 (25.74%) | 63 (22.03%) |  |  |  |  |
| Year 4 | 288 (20.76%) | 66 (23.08%) |  |  |  |  |
| Major group |  |  | χ²(3) = 4.093 | .252 | .252 | V = .049 |
| STEM | 557 (40.16%) | 98 (34.27%) |  |  |  |  |
| Humanities/social sciences | 444 (32.01%) | 103 (36.01%) |  |  |  |  |
| Medicine/health | 243 (17.52%) | 50 (17.48%) |  |  |  |  |
| Arts/design | 143 (10.31%) | 35 (12.24%) |  |  |  |  |
| Subjective socioeconomic status |  |  | χ²(2) = 4.549 | .103 | .225 | V = .052 |
| Low | 270 (19.47%) | 69 (24.13%) |  |  |  |  |
| Middle | 800 (57.68%) | 164 (57.34%) |  |  |  |  |
| High | 317 (22.86%) | 53 (18.53%) |  |  |  |  |
| BMI category |  |  | χ²(2) = 6.021 | .049 | .197 | V = .060 |
| Underweight | 200 (14.42%) | 31 (10.84%) |  |  |  |  |
| Normal weight | 1005 (72.46%) | 204 (71.33%) |  |  |  |  |
| Overweight/obesity | 182 (13.12%) | 51 (17.83%) |  |  |  |  |

Note. Complete follow-up denotes participation at all three waves; incomplete follow-up denotes at least one missing follow-up wave. Continuous scores are M (SD); categorical entries are n (% within group). Baseline PPF was available for 1,386 complete-follow-up participants and all 286 incomplete-follow-up participants. Hedges’ g is incomplete minus complete; positive values indicate higher baseline scores in the incomplete group. Cramér’s V accompanies Pearson chi-square tests. Holm adjustment (Holm, 1979) was applied within the three continuous and five categorical comparisons separately. ESE = exercise self-efficacy; EEI = extracurricular exercise involvement; PPF = perceived physical fitness; CI = confidence interval. BMI = body mass index; STEM = science, technology, engineering, and mathematics.

**Table S2**

Multivariable logistic regression of complete three-wave participation

| **Baseline predictor** | **B** | **SE** | **OR** | **95% CI for OR** | **p** | **Holm p** |
| --- | --- | --- | --- | --- | --- | --- |
| ESE at T1 (per SD) | −.231 | .076 | 0.794 | [.684, .922] | .003 | .036 |
| EEI at T1 (per SD) | −.129 | .077 | 0.879 | [.756, 1.022] | .093 | .742 |
| PPF at T1 (per SD) | −.066 | .076 | 0.936 | [.807, 1.085] | .381 | .985 |
| Female versus male | −.405 | .136 | 0.667 | [.511, .870] | .003 | .037 |
| Year 2 versus Year 1 | −.364 | .180 | 0.695 | [.488, .990] | .044 | .435 |
| Year 3 versus Year 1 | .024 | .194 | 1.025 | [.701, 1.498] | .900 | .985 |
| Year 4 versus Year 1 | −.221 | .192 | 0.801 | [.550, 1.169] | .250 | .985 |
| Humanities/social sciences versus STEM | −.294 | .158 | 0.745 | [.547, 1.015] | .062 | .561 |
| Medicine/health versus STEM | −.239 | .194 | 0.787 | [.538, 1.151] | .217 | .985 |
| Arts/design versus STEM | −.353 | .223 | 0.702 | [.454, 1.087] | .113 | .790 |
| Middle versus low subjective SES | .229 | .163 | 1.258 | [.913, 1.733] | .161 | .964 |
| High versus low subjective SES | .429 | .204 | 1.536 | [1.030, 2.291] | .035 | .390 |
| Normal weight versus underweight | −.275 | .213 | 0.760 | [.501, 1.153] | .197 | .985 |
| Overweight/obesity versus underweight | −.564 | .255 | 0.569 | [.345, .938] | .027 | .326 |

Note. Outcome: 1 = participation at all three waves; 0 = at least one missing follow-up. N = 1,672, including 1,386 complete participants and 286 with incomplete follow-up; one participant with missing baseline PPF was excluded from this regression. ESE, EEI, and PPF were standardized before fitting the model, so their odds ratios are per one SD. Holm adjustment (Holm, 1979) covered the 14 predictor coefficients. Likelihood-ratio χ²(14) = 53.168, p < .001; Nagelkerke R² = .052; AUC = .634. Confidence intervals are unadjusted 95% Wald intervals. ESE = exercise self-efficacy; EEI = extracurricular exercise involvement; PPF = perceived physical fitness; CI = confidence interval. B = log-odds coefficient; OR = odds ratio; SES = socioeconomic status; BMI = body mass index; STEM = science, technology, engineering, and mathematics; AUC = area under the receiver-operating-characteristic curve.

**Table S3**

Wave-specific confirmatory factor analyses

| **Construct** | **Wave** | **N** | **Scaled χ² (df)** | **p** | **CFI** | **TLI** | **RMSEA [90% CI]** | **SRMR** |
| --- | --- | --- | --- | --- | --- | --- | --- | --- |
| ESE | T1 | 1673 | 28.739 (34) | .723 | 1.000 | 1.001 | .000 [.000, .014] | .011 |
| ESE | T2 | 1543 | 41.306 (34) | .182 | .999 | .998 | .012 [.000, .023] | .012 |
| ESE | T3 | 1455 | 26.637 (34) | .812 | 1.000 | 1.002 | .000 [.000, .012] | .010 |
| EEI | T1 | 1673 | 142.074 (164) | .891 | 1.000 | 1.002 | .000 [.000, .005] | .013 |
| EEI | T2 | 1543 | 154.260 (164) | .696 | 1.000 | 1.001 | .000 [.000, .009] | .014 |
| EEI | T3 | 1455 | 148.408 (164) | .803 | 1.000 | 1.002 | .000 [.000, .008] | .015 |
| PPF | T1 | 1673 | 2.434 (5) | .786 | 1.000 | 1.003 | .000 [.000, .022] | .005 |
| PPF | T2 | 1543 | 5.508 (5) | .357 | 1.000 | .999 | .008 [.000, .037] | .008 |
| PPF | T3 | 1455 | 10.436 (5) | .064 | .997 | .994 | .027 [.000, .051] | .011 |

Note. Models used MLR with FIML and treated five-category items as approximately continuous. ESE specified two correlated factors; EEI specified four correlated factors; PPF specified a single diagnostic factor for shared variance among its five domain indicators. CFA sample sizes include participants with partial item responses, including PPF cases below the composite-scoring threshold. All nine models converged normally. TLI values above 1 are shown without truncation. ESE = exercise self-efficacy; EEI = extracurricular exercise involvement; PPF = perceived physical fitness; CI = confidence interval. MLR = robust maximum likelihood; FIML = full-information maximum likelihood; CFI = comparative fit index; TLI = Tucker–Lewis index; RMSEA = root mean square error of approximation; SRMR = standardized root mean square residual.

**Table S4**

Factor loadings, composite reliability, and average variance extracted

| **Construct** | **Wave** | **Factor** | **Items** | **Standardized loading range** | **CR** | **AVE** |
| --- | --- | --- | --- | --- | --- | --- |
| ESE | T1 | Situational motivation | 6 | .587–.723 | .824 | .440 |
| ESE | T1 | Subjective support | 4 | .665–.785 | .823 | .538 |
| ESE | T2 | Situational motivation | 6 | .585–.760 | .835 | .460 |
| ESE | T2 | Subjective support | 4 | .613–.781 | .795 | .494 |
| ESE | T3 | Situational motivation | 6 | .650–.801 | .859 | .505 |
| ESE | T3 | Subjective support | 4 | .641–.773 | .800 | .501 |
| EEI | T1 | Vigor-persistence | 6 | .635–.748 | .848 | .483 |
| EEI | T1 | Absorption and satisfaction | 6 | .651–.801 | .868 | .524 |
| EEI | T1 | Value cognition | 5 | .642–.787 | .846 | .525 |
| EEI | T1 | Participation autonomy | 3 | .765–.766 | .810 | .586 |
| EEI | T2 | Vigor-persistence | 6 | .609–.726 | .836 | .460 |
| EEI | T2 | Absorption and satisfaction | 6 | .624–.831 | .866 | .520 |
| EEI | T2 | Value cognition | 5 | .651–.800 | .848 | .528 |
| EEI | T2 | Participation autonomy | 3 | .765–.795 | .822 | .606 |
| EEI | T3 | Vigor-persistence | 6 | .605–.724 | .828 | .446 |
| EEI | T3 | Absorption and satisfaction | 6 | .613–.805 | .853 | .493 |
| EEI | T3 | Value cognition | 5 | .613–.797 | .843 | .520 |
| EEI | T3 | Participation autonomy | 3 | .809–.837 | .866 | .682 |
| PPF | T1 | PPF diagnostic factor | 5 | .618–.683 | .777 | .411 |
| PPF | T2 | PPF diagnostic factor | 5 | .593–.752 | .794 | .437 |
| PPF | T3 | PPF diagnostic factor | 5 | .589–.804 | .817 | .474 |

Note. CR = (Σλ)² / [(Σλ)² + Σθ]; AVE = Σλ² / (Σλ² + Σθ), where λ is a standardized loading and θ is the corresponding residual variance. Model-based omega under these congeneric specifications equals the reported CR. ESE factor correlations were .803, .801, and .753 at T1, T2, and T3, respectively; correlations among EEI factors ranged from .657 to .699, .645 to .676, and .568 to .706. Several AVE values were below .50. The strong ESE factor correlations indicate substantial dimensional overlap. PPF values describe the single-factor diagnostic and do not establish interchangeability of the five fitness domains. ESE = exercise self-efficacy; EEI = extracurricular exercise involvement; PPF = perceived physical fitness; CI = confidence interval. CR = composite reliability; AVE = average variance extracted.

**Table S5**

Longitudinal measurement invariance model fit

| **Construct** | **Model** | **Scaled χ² (df)** | **p** | **CFI** | **TLI** | **RMSEA [90% CI]** | **SRMR** |
| --- | --- | --- | --- | --- | --- | --- | --- |
| ESE | Configural | 354.568 (360) | .571 | 1.000 | 1.000 | .000 [.000, .008] | .016 |
| ESE | Metric | 393.543 (376) | .257 | .999 | .999 | .005 [.000, .010] | .021 |
| ESE | Scalar | 769.798 (392) | < .001 | .979 | .976 | .024 [.021, .026] | .029 |
| ESE | Partial scalar | 564.272 (390) | < .001 | .990 | .989 | .016 [.013, .019] | .025 |
| EEI | Configural | 1584.889 (1584) | .489 | 1.000 | 1.000 | .001 [.000, .006] | .017 |
| EEI | Metric | 1633.078 (1616) | .378 | 1.000 | 1.000 | .003 [.000, .007] | .019 |
| EEI | Scalar | 1879.529 (1648) | < .001 | .994 | .994 | .009 [.007, .011] | .021 |
| PPF | Configural | 75.601 (72) | .363 | .999 | .999 | .005 [.000, .015] | .014 |
| PPF | Metric | 125.923 (80) | < .001 | .993 | .991 | .019 [.012, .025] | .031 |
| PPF | Scalar | 198.870 (88) | < .001 | .984 | .981 | .027 [.022, .033] | .037 |

Note. N = 1,673 for all models. Corresponding item residuals were correlated across the three waves. Metric models constrained corresponding factor loadings; scalar models additionally constrained item intercepts. The ESE partial scalar model freed only the intercept of subjective support Item 1 at T3 (ESE_D2_I01_T3) and situational motivation Item 6 at T1 (ESE_D1_I06_T1). All other cross-wave intercept constraints and all loading constraints were retained. EEI and PPF retained full scalar constraints under the approximate-fit-change criteria. ESE = exercise self-efficacy; EEI = extracurricular exercise involvement; PPF = perceived physical fitness; CI = confidence interval. CFI = comparative fit index; TLI = Tucker–Lewis index; RMSEA = root mean square error of approximation; SRMR = standardized root mean square residual.

**Table S6**

Comparisons of longitudinal measurement invariance constraints

| **Construct** | **Comparison** | **Scaled Δχ² (Δdf)** | **p** | **ΔCFI** | **ΔRMSEA** | **ΔSRMR** | **Approximate invariance** |
| --- | --- | --- | --- | --- | --- | --- | --- |
| ESE | Metric − configural | 45.146 (16) | < .001 | −.001 | .005 | .005 | Supported |
| ESE | Scalar − metric | 386.401 (16) | < .001 | −.020 | .019 | .008 | Not supported |
| ESE | Partial scalar − metric | 174.918 (14) | < .001 | −.009 | .011 | .004 | Supported |
| EEI | Metric − configural | 52.795 (32) | .012 | .000 | .002 | .002 | Supported |
| EEI | Scalar − metric | 251.850 (32) | < .001 | −.006 | .006 | .002 | Supported |
| PPF | Metric − configural | 58.802 (8) | < .001 | −.006 | .014 | .017 | Supported |
| PPF | Scalar − metric | 74.947 (8) | < .001 | −.009 | .008 | .006 | Supported |

Note. Differences are the more constrained model minus its comparison model. Invariance was evaluated using CFI decreases ≤ .010 and RMSEA increases ≤ .015, together with SRMR increases ≤ .030 for metric constraints and ≤ .010 for scalar constraints (Chen, 2007). MLR-scaled likelihood-ratio difference tests are reported separately; several reject exact equality despite acceptable approximate-fit changes. The ESE partial scalar comparison uses the metric model as its reference and the two freed intercepts described in Table S5. ESE = exercise self-efficacy; EEI = extracurricular exercise involvement; PPF = perceived physical fitness; CI = confidence interval. CFI = comparative fit index; RMSEA = root mean square error of approximation; SRMR = standardized root mean square residual.

**Table S7**

Random-intercept and within-person variance estimates

| **Construct** | **Variance component** | **Occasion** | **Estimate** | **SE** | **95% CI** | **p** |
| --- | --- | --- | --- | --- | --- | --- |
| ESE | Random intercept | Across waves | .332 | .027 | [.278, .386] | < .001 |
| EEI | Random intercept | Across waves | .266 | .026 | [.216, .316] | < .001 |
| PPF | Random intercept | Across waves | .395 | .031 | [.335, .456] | < .001 |
| ESE | Within-person variance | T1 | .430 | .028 | [.376, .484] | < .001 |
| EEI | Within-person variance | T1 | .383 | .026 | [.332, .434] | < .001 |
| PPF | Within-person variance | T1 | .402 | .030 | [.344, .461] | < .001 |
| ESE | Innovation variance | T2 | .411 | .024 | [.364, .458] | < .001 |
| EEI | Innovation variance | T2 | .337 | .018 | [.302, .372] | < .001 |
| PPF | Innovation variance | T2 | .428 | .023 | [.382, .474] | < .001 |
| ESE | Innovation variance | T3 | .422 | .019 | [.385, .459] | < .001 |
| EEI | Innovation variance | T3 | .328 | .018 | [.293, .363] | < .001 |
| PPF | Innovation variance | T3 | .476 | .021 | [.435, .517] | < .001 |

Note. Estimates are unstandardized and use MLR-based standard errors from the free-lag RI-CLPM (N = 1,673). Random-intercept variances represent stable between-person differences. T1 within-person variances are unconditional initial variances. T2 and T3 values are innovation variances conditional on all three prior-wave components; they are not total within-person variances. Descriptive ICCs in main Table 2 come from separate univariate random-intercept models with fixed wave effects and quantify a different variance decomposition. ESE = exercise self-efficacy; EEI = extracurricular exercise involvement; PPF = perceived physical fitness; CI = confidence interval.

**Table S8**

Between-person and within-wave associations in the free-lag RI-CLPM

| **Level or occasion** | **Pair** | **Covariance** | **SE of covariance** | **Correlation** | **SE of correlation** | **95% CI for correlation** | **p** |
| --- | --- | --- | --- | --- | --- | --- | --- |
| Random intercepts | ESE–EEI | .178 | .021 | .600 | .045 | [.512, .688] | < .001 |
| Random intercepts | ESE–PPF | .224 | .023 | .618 | .046 | [.528, .707] | < .001 |
| Random intercepts | EEI–PPF | .181 | .023 | .558 | .046 | [.469, .647] | < .001 |
| T1 within-person | ESE–EEI | .080 | .022 | .198 | .047 | [.106, .291] | < .001 |
| T1 within-person | ESE–PPF | .061 | .023 | .147 | .050 | [.048, .245] | .004 |
| T1 within-person | EEI–PPF | .106 | .023 | .269 | .048 | [.174, .364] | < .001 |
| T2 innovations | ESE–EEI | .086 | .017 | .232 | .042 | [.149, .315] | < .001 |
| T2 innovations | ESE–PPF | .089 | .019 | .213 | .042 | [.130, .296] | < .001 |
| T2 innovations | EEI–PPF | .077 | .017 | .203 | .041 | [.122, .285] | < .001 |
| T3 innovations | ESE–EEI | .082 | .014 | .220 | .036 | [.150, .289] | < .001 |
| T3 innovations | ESE–PPF | .057 | .016 | .128 | .034 | [.060, .195] | < .001 |
| T3 innovations | EEI–PPF | .088 | .014 | .223 | .034 | [.157, .289] | < .001 |

Note. N = 1,673. Covariances are unstandardized; correlations and their standard errors are from STDYX output. T1 associations refer to the initial within-person components; T2 and T3 associations refer to innovation residuals conditional on the preceding wave. Reported p values concern the correlations. ESE = exercise self-efficacy; EEI = extracurricular exercise involvement; PPF = perceived physical fitness; CI = confidence interval.

**Table S9**

Scaled comparisons of lagged-path equality constraints

| **Restricted model** | **Comparison model** | **Δdf** | **Scaling correction** | **Scaled Δχ²** | **p** |
| --- | --- | --- | --- | --- | --- |
| Equal autoregressive paths | Free lags | 3 | 0.9666 | 6.249 | .100 |
| Equal cross-lagged paths | Free lags | 6 | 0.9983 | 14.698 | .023 |
| All lagged paths equal | Free lags | 9 | 1.0036 | 22.425 | .008 |
| All lagged paths equal | Equal autoregressive paths | 6 | 1.0221 | 16.110 | .013 |
| All lagged paths equal | Equal cross-lagged paths | 3 | 1.0141 | 7.723 | .052 |

Note. All models used the same nine composite scores and N = 1,673 with MLR/FIML. Equal autoregressive paths impose three between-interval restrictions; equal cross-lagged paths impose six; the fully equal-lag model imposes all nine. Scaled likelihood-ratio differences use the models’ log-likelihoods, free-parameter counts, and H0 scaling correction factors. AIC and BIC for the primary free-lag and fully equal-lag models are provided in main Table 3. ESE = exercise self-efficacy; EEI = extracurricular exercise involvement; PPF = perceived physical fitness; CI = confidence interval. MLR = robust maximum likelihood; FIML = full-information maximum likelihood.

**Table S10**

Between-interval differences in autoregressive and cross-lagged coefficients

| **Path** | **b₁₂** | **b₂₃** | **β₁₂** | **β₂₃** | **Δb** | **SE of Δb** | **Approx. 95% CI for Δb** | **Wald χ²(1)** | **p** | **Holm p** |
| --- | --- | --- | --- | --- | --- | --- | --- | --- | --- | --- |
| ESE → ESE | .150 | .197 | .151 | .190 | .047 | .048 | [−.047, .141] | 0.945 | .331 | 1.000 |
| EEI → EEI | .277 | .188 | .280 | .191 | −.089 | .043 | [−.173, −.005] | 4.239 | .040 | .320 |
| PPF → PPF | .233 | .187 | .217 | .176 | −.046 | .048 | [−.140, .048] | 0.918 | .338 | 1.000 |
| EEI → ESE | .065 | .143 | .061 | .129 | .078 | .057 | [−.034, .190] | 1.858 | .173 | 1.000 |
| PPF → ESE | .027 | .051 | .026 | .052 | .024 | .055 | [−.084, .132] | 0.194 | .659 | 1.000 |
| ESE → EEI | .093 | .166 | .100 | .179 | .073 | .048 | [−.021, .167] | 2.341 | .126 | .882 |
| PPF → EEI | .007 | .056 | .008 | .063 | .049 | .052 | [−.053, .151] | 0.874 | .350 | 1.000 |
| ESE → PPF | .061 | .003 | .059 | .003 | −.058 | .054 | [−.164, .048] | 1.153 | .283 | 1.000 |
| EEI → PPF | .112 | .242 | .101 | .205 | .130 | .058 | [.016, .244] | 4.964 | .026 | .234 |

Note. The joint Wald test of all nine equality restrictions was χ²(9) = 23.372, p = .0054. The subscripts 12 and 23 denote the T1-to-T2 and T2-to-T3 intervals. Differences are Δb = b₂₃ − b₁₂ and are tested on unstandardized parameters, accounting for their sampling covariance. β denotes the corresponding STDYX estimate. Individual confidence intervals are unadjusted approximations from Δb ± 1.96 SE; they are not simultaneous intervals. Holm adjustment (Holm, 1979) applies to the nine individual tests. No individual difference survived this adjustment. ESE = exercise self-efficacy; EEI = extracurricular exercise involvement; PPF = perceived physical fitness; CI = confidence interval.

**Table S11**

Fit of primary and sensitivity RI-CLPMs

| **Model** | **N** | **k** | **Scaled χ² (df)** | **p** | **CFI** | **TLI** | **RMSEA [90% CI]** | **SRMR** |
| --- | --- | --- | --- | --- | --- | --- | --- | --- |
| Primary free-lag | 1673 | 51 | 6.667 (3) | .083 | .999 | .991 | .027 [.000, .055] | .008 |
| Demographics: RI + T1 within | 1673 | 117 | 44.027 (36) | .168 | .998 | .994 | .012 [.000, .022] | .010 |
| Demographics: RI only | 1673 | 84 | 98.655 (69) | .011 | .994 | .989 | .016 [.008, .023] | .013 |
| Complete nine-score cases | 1383 | 51 | 6.746 (3) | .080 | .999 | .990 | .030 [.000, .061] | .008 |
| Attrition-informed: female | 1673 | 57 | 8.989 (6) | .174 | .999 | .995 | .017 [.000, .039] | .008 |
| Additional ESE_T1 → PPF_T3 | 1673 | 52 | 6.400 (2) | .041 | .999 | .984 | .036 [.006, .069] | .008 |

Note. All models used MLR. RI = random intercept; T1 within = initial within-person components; k = number of free parameters. The demographic models used 11 dummy variables for gender, academic year, major, subjective socioeconomic status, and BMI category. The attrition-informed model included the female indicator as a predictor of all three RIs and all three T1 within-person components. Compared with the primary model, maximum absolute changes in the 18 standardized lagged estimates were .022 for demographics predicting RI + T1 within, .006 for RI-only adjustment, .023 for complete cases, .004 for female adjustment, and .022 for the additional long-lag path. All models retained the same p < .05 classification for all 18 paths. Two near-zero nonsignificant coefficients changed sign under full demographic adjustment and one under the additional long-lag path. ESE = exercise self-efficacy; EEI = extracurricular exercise involvement; PPF = perceived physical fitness; CI = confidence interval. CFI = comparative fit index; TLI = Tucker–Lewis index; RMSEA = root mean square error of approximation; SRMR = standardized root mean square residual.

**Table S12**

Lagged associations with full demographic adjustment

| **Path** | **Primary β** | **Primary 95% CI** | **Primary p** | **Adjusted β** | **Adjusted 95% CI** | **Adjusted p** |
| --- | --- | --- | --- | --- | --- | --- |
| ESE_T1 → ESE_T2 | .151 | [.047, .255] | .004 | .143 | [.037, .248] | .008 |
| EEI_T1 → ESE_T2 | .061 | [−.030, .153] | .189 | .050 | [−.043, .143] | .292 |
| PPF_T1 → ESE_T2 | .026 | [−.074, .126] | .607 | .027 | [−.074, .128] | .601 |
| ESE_T1 → EEI_T2 | .100 | [.014, .185] | .023 | .092 | [.003, .182] | .043 |
| EEI_T1 → EEI_T2 | .280 | [.189, .372] | < .001 | .267 | [.171, .362] | < .001 |
| PPF_T1 → EEI_T2 | .008 | [−.088, .103] | .875 | −.007 | [−.106, .093] | .896 |
| ESE_T1 → PPF_T2 | .059 | [−.025, .142] | .169 | .063 | [−.021, .147] | .139 |
| EEI_T1 → PPF_T2 | .101 | [.010, .192] | .029 | .092 | [.002, .183] | .046 |
| PPF_T1 → PPF_T2 | .217 | [.113, .320] | < .001 | .226 | [.123, .329] | < .001 |
| ESE_T2 → ESE_T3 | .190 | [.093, .287] | < .001 | .172 | [.074, .269] | .001 |
| EEI_T2 → ESE_T3 | .129 | [.041, .217] | .004 | .121 | [.030, .212] | .009 |
| PPF_T2 → ESE_T3 | .052 | [−.033, .136] | .231 | .058 | [−.028, .144] | .186 |
| ESE_T2 → EEI_T3 | .179 | [.093, .265] | < .001 | .177 | [.091, .264] | < .001 |
| EEI_T2 → EEI_T3 | .191 | [.086, .295] | < .001 | .169 | [.063, .275] | .002 |
| PPF_T2 → EEI_T3 | .063 | [−.022, .149] | .148 | .070 | [−.017, .157] | .114 |
| ESE_T2 → PPF_T3 | .003 | [−.080, .086] | .945 | −.006 | [−.089, .077] | .886 |
| EEI_T2 → PPF_T3 | .205 | [.114, .295] | < .001 | .191 | [.098, .283] | < .001 |
| PPF_T2 → PPF_T3 | .176 | [.077, .275] | < .001 | .179 | [.080, .279] | < .001 |

Note. The adjusted model includes all 11 baseline demographic dummy variables as predictors of both the three random intercepts and the three initial within-person components (117 free parameters; N = 1,673). Values are STDYX estimates with MLR-based Wald intervals. Confidence intervals for the primary model use unrounded estimates and standard errors; adjusted intervals are the Mplus 95% intervals. Coefficient comparisons describe changes in path magnitude and uncertainty. ESE = exercise self-efficacy; EEI = extracurricular exercise involvement; PPF = perceived physical fitness; CI = confidence interval.

**Table S13**

Lagged associations under RI-only and attrition-informed adjustment

| **Path** | **RI-only β** | **RI-only 95% CI** | **RI-only p** | **Female-adjusted β** | **Female-adjusted 95% CI** | **Female-adjusted p** |
| --- | --- | --- | --- | --- | --- | --- |
| ESE_T1 → ESE_T2 | .151 | [.048, .254] | .004 | .152 | [.049, .256] | .004 |
| EEI_T1 → ESE_T2 | .056 | [−.036, .148] | .232 | .060 | [−.032, .151] | .201 |
| PPF_T1 → ESE_T2 | .030 | [−.070, .129] | .562 | .029 | [−.071, .129] | .570 |
| ESE_T1 → EEI_T2 | .098 | [.012, .184] | .025 | .100 | [.014, .185] | .022 |
| EEI_T1 → EEI_T2 | .276 | [.183, .368] | < .001 | .280 | [.188, .371] | < .001 |
| PPF_T1 → EEI_T2 | .005 | [−.091, .101] | .919 | .008 | [−.088, .103] | .872 |
| ESE_T1 → PPF_T2 | .061 | [−.022, .145] | .151 | .061 | [−.023, .144] | .154 |
| EEI_T1 → PPF_T2 | .099 | [.008, .190] | .033 | .099 | [.008, .190] | .033 |
| PPF_T1 → PPF_T2 | .216 | [.113, .318] | < .001 | .221 | [.117, .324] | < .001 |
| ESE_T2 → ESE_T3 | .187 | [.090, .284] | < .001 | .191 | [.095, .287] | < .001 |
| EEI_T2 → ESE_T3 | .126 | [.037, .215] | .005 | .128 | [.040, .216] | .004 |
| PPF_T2 → ESE_T3 | .052 | [−.033, .137] | .228 | .053 | [−.031, .138] | .218 |
| ESE_T2 → EEI_T3 | .179 | [.092, .265] | < .001 | .180 | [.094, .265] | < .001 |
| EEI_T2 → EEI_T3 | .185 | [.079, .290] | .001 | .190 | [.086, .294] | < .001 |
| PPF_T2 → EEI_T3 | .064 | [−.022, .150] | .145 | .063 | [−.022, .149] | .148 |
| ESE_T2 → PPF_T3 | .000 | [−.083, .083] | .998 | .003 | [−.080, .086] | .950 |
| EEI_T2 → PPF_T3 | .203 | [.112, .294] | < .001 | .204 | [.114, .295] | < .001 |
| PPF_T2 → PPF_T3 | .175 | [.076, .273] | .001 | .177 | [.078, .276] | < .001 |

Note. Both models used N = 1,673. The RI-only model includes all 11 demographic dummy variables as predictors of the random intercepts only (84 free parameters). The attrition-informed model includes the female indicator as a predictor of the three RIs and the three T1 within-person components (57 free parameters); baseline ESE is already included in the longitudinal structure. This covariate analysis quantifies sensitivity to an observed attrition correlate and does not verify the MAR assumption. Values are STDYX estimates with MLR-based Wald intervals. ESE = exercise self-efficacy; EEI = extracurricular exercise involvement; PPF = perceived physical fitness; CI = confidence interval. RI = random intercept; MAR = missing at random.

**Table S14**

Lagged associations with an additional T1-to-T3 ESE-to-PPF path

| **Path** | **b** | **SE of b** | **β** | **SE of β** | **95% CI for β** | **p** |
| --- | --- | --- | --- | --- | --- | --- |
| ESE_T1 → ESE_T2 | .148 | .055 | .149 | .054 | [.043, .254] | .006 |
| EEI_T1 → ESE_T2 | .066 | .049 | .063 | .047 | [−.029, .155] | .178 |
| PPF_T1 → ESE_T2 | .007 | .062 | .007 | .060 | [−.111, .125] | .907 |
| ESE_T1 → EEI_T2 | .092 | .041 | .098 | .044 | [.012, .185] | .026 |
| EEI_T1 → EEI_T2 | .277 | .049 | .280 | .047 | [.188, .372] | < .001 |
| PPF_T1 → EEI_T2 | .005 | .047 | .005 | .049 | [−.090, .100] | .919 |
| ESE_T1 → PPF_T2 | .038 | .059 | .037 | .057 | [−.074, .148] | .513 |
| EEI_T1 → PPF_T2 | .108 | .051 | .099 | .047 | [.006, .191] | .036 |
| PPF_T1 → PPF_T2 | .234 | .058 | .218 | .053 | [.114, .322] | < .001 |
| ESE_T2 → ESE_T3 | .200 | .050 | .193 | .049 | [.096, .289] | < .001 |
| EEI_T2 → ESE_T3 | .146 | .049 | .132 | .045 | [.043, .220] | .003 |
| PPF_T2 → ESE_T3 | .037 | .048 | .038 | .049 | [−.058, .133] | .439 |
| ESE_T2 → EEI_T3 | .167 | .040 | .180 | .044 | [.094, .265] | < .001 |
| EEI_T2 → EEI_T3 | .188 | .052 | .190 | .053 | [.086, .294] | < .001 |
| PPF_T2 → EEI_T3 | .054 | .038 | .061 | .044 | [−.024, .147] | .159 |
| ESE_T2 → PPF_T3 | −.018 | .058 | −.016 | .052 | [−.118, .086] | .762 |
| EEI_T2 → PPF_T3 | .244 | .054 | .207 | .047 | [.114, .300] | < .001 |
| PPF_T2 → PPF_T3 | .187 | .053 | .177 | .051 | [.076, .277] | .001 |
| ESE_T1 → PPF_T3 | −.035 | .056 | −.032 | .051 | [−.131, .068] | .534 |

Note. N = 1,673. The model retains all 18 adjacent-wave paths and adds a path from within-person ESE at T1 to within-person PPF at T3. The added path spans approximately 16 weeks and uses the same predictor and outcome occasions as the focal three-wave indirect association. Its unstandardized coefficient was −.035 (SE = .056, p = .534), and its STDYX estimate was −.032, 95% CI [−.131, .068]. All entries use the MLR model output; reported p values concern the standardized estimates. ESE = exercise self-efficacy; EEI = extracurricular exercise involvement; PPF = perceived physical fitness; CI = confidence interval. b = unstandardized coefficient; β = STDYX coefficient.

**Table S15**

Specific three-wave indirect associations across sensitivity models

| **Model** | **N** | **b** | **SE of b** | **Wald p for b** | **βind** | **SE of βind** | **Wald p for βind** | **95% Wald CI for βind** |
| --- | --- | --- | --- | --- | --- | --- | --- | --- |
| Primary free-lag | 1,673 | .023 | .012 | .055 | .020 | .011 | .057 | [−.001, .041] |
| Demographics: RI + T1 within | 1,673 | .019 | .011 | .087 | .018 | .010 | .090 | [−.003, .038] |
| Demographics: RI only | 1,673 | .022 | .012 | .059 | .020 | .011 | .062 | [−.001, .041] |
| Attrition-informed: female | 1,673 | .022 | .012 | .055 | .020 | .011 | .057 | [−.001, .041] |
| Complete nine-score cases | 1,383 | .023 | .012 | .064 | .021 | .011 | .066 | [−.001, .042] |
| Additional ESE_T1 → PPF_T3 | 1,673 | .023 | .012 | .065 | .020 | .011 | .068 | [−.002, .042] |

Note. The specific pathway is within-person ESE_T1 → EEI_T2 → PPF_T3 in every model. b denotes the unstandardized product, and βind denotes its STDYX standardized estimate. All standard errors, p values, and intervals in this table use MLR-based normal-theory inference. Bootstrap intervals for the primary model are reported in main Table 5. Primary unrounded estimates are b = .022501571 and βind = .020382062. ESE = exercise self-efficacy; EEI = extracurricular exercise involvement; PPF = perceived physical fitness; CI = confidence interval. RI = random intercept; T1 within = initial within-person components.

**References**

Chen, F. F. (2007). Sensitivity of goodness of fit indexes to lack of measurement invariance. Structural Equation Modeling: A Multidisciplinary Journal, 14(3), 464–504. https://doi.org/10.1080/10705510701301834

Holm, S. (1979). A simple sequentially rejective multiple test procedure. Scandinavian Journal of Statistics, 6(2), 65–70. https://www.jstor.org/stable/4615733
